# Clathrin Light Chain B Drives Hepatocellular Carcinoma Progression Through Dual Mechanisms: Small Extracellular Vesicle‐Mediated Angiogenesis and the NF‐κB–PCLAF Signaling Axis

**DOI:** 10.1002/advs.202508613

**Published:** 2025-08-18

**Authors:** Xiaoke Sun, Junchen Guo, Ning Zhao, Guanghua Cui, Yun Bai, Meijuan Ding, Yi Xu, Yu Yang

**Affiliations:** ^1^ Department of Internal Medical Oncology Second Affiliated Hospital of Harbin Medical University No. 246 Xuefu ROAD Harbin 150086 P. R. China; ^2^ Department of Cardiology Second Affiliated Hospital of Harbin Medical University No. 246 Xuefu ROAD Harbin 150086 P. R. China; ^3^ Department of Hepatopancreatobiliary Surgery Second Affiliated Hospital of Harbin Medical University No. 246 Xuefu ROAD Harbin 150086 P. R. China; ^4^ State Key Laboratory of Oncology in South China Cancer Center of Sun Yat‐sen University Guangzhou Guangdong P. R. China; ^5^ Department of Pathology The Li Ka Shing Faculty of Medicine at The University of Hong Kong Hong Kong 999077 P. R. China

**Keywords:** clathrin light chain B, hepatocellular carcinoma, intercellular communication, small extracellular vesicles, vascular permeability

## Abstract

Clathrin light chain B (CLTB) is one of the three light chain subunits of the clathrin complex. This study aims to elucidate the role of CLTB in the pathogenesis of hepatocellular carcinoma (HCC) and its clinical implications. Clinical and bioinformatic analyses reveal marked CLTB overexpression in HCC tissues. Genetic silencing of CLTB suppresses HCC cell proliferation, migration, and invasion, whereas its overexpression exacerbates malignant phenotypes. Mechanistically, CLTB activates NF‐κB signaling to upregulate PCNA clamp‐associated factor (PCLAF), thereby promoting small extracellular vesicle (sEV) uptake. Given that clathrin‐mediated endocytosis is the key mechanism for sEV uptake, this study further investigated the functional implications of CLTB‐enriched sEVs in tumor vascular remodeling. sEV‐CLTB promotes endothelial angiogenesis, disrupts vascular integrity, and induces pulmonary vascular leakage by binding SH3 domain‐containing kinase‐binding protein 1 (SH3KBP1) and then inhibiting SH3KBP1 ubiquitination degradation. In patient‐derived xenograft (PDX) models, combined therapy of clathrin inhibitor (chlorpromazine) or SH3KBP1 silencing with sorafenib suppresses tumor growth and reduces microvascular density. This study demonstrates that CLTB promotes HCC progression through the NF‐κB–PCLAF signaling axis and sEV‐mediated vascular remodeling, providing a mechanistic foundation for developing combination therapies targeting CLTB.

## Graphical abstract

Clathrin Light Chain B (CLTB) drives hepatocellular carcinoma progression through dual pathways: intracellularly, it activates the NF‐κB–PCLAF axis to enhance small extracellular vesicle (sEV) uptake and tumor proliferation; extracellularly, CLTB‐enriched sEVs bind SH3KBP1 to inhibit its degradation, inducing angiogenesis and vascular leakage. Targeting CLTB or SH3KBP1 synergizes with sorafenib to suppress tumor growth in vivo.

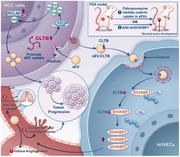

## 1 Introduction

Among malignant tumors, liver cancer is one of the most clinically challenging solid tumors and poses a serious threat to public health worldwide.^[^ ^1^, ^2^ ^]^ Approximately 90% of liver cancer cases are hepatocellular carcinoma (HCC), a subtype characterized by a rich vascular network, irregular branching, and leakages.^[^ ^3^ ^]^ Neo‐angiogenesis drives the endothelial cell proliferation process, sustains the metabolic demands of cancer cells, and promotes invasion by establishing a disordered microcirculatory system.^[^ ^4^ ^]^ Despite the significant progress in systemic therapy, including targeted therapies and immune checkpoint inhibitors, the high vascular density in HCC remains a major barrier to effective treatment. Most patients are diagnosed at intermediate‐advanced stages, often presenting with portal vein invasion or extrahepatic metastasis, which leads to a poor prognosis.^[^ ^5^ ^]^

Small extracellular vesicles (sEVs), cell‐secreted lipid bilayer membrane‐bound vesicles, serve as key mediators of intercellular communication under physiological and pathological conditions.^[^ ^6^ ^]^ sEVs are crucial for the development and spread of various malignant tumors. They target specific organs to remodel the tumor microenvironment, modulate interactions between tumor and normal tissues, and influence cancer‐related processes such as cell proliferation.^[^ ^7^ ^]^ Notably, tumor‐cell‐derived sEVs promote angiogenesis and exacerbate vascular leakage.^[^ ^8^ ^]^

Clathrin light chain B (CLTB) is one of the three light chain subunits constituting the clathrin complex. Clathrin forms structural components of clathrin‐coated pits essential for receptor‐mediated endocytosis.^[^ ^9^ ^]^ Clathrin‐mediated endocytosis (CME) is the key mechanism for cellular uptake of sEVs, and is crucial for regulating cellular communication, motility polarity, membrane protein turnover, and nutrient uptake.^[^ ^10^, ^11^, ^12^, ^13^ ^]^ Besides endocytosis, clathrin can also influence actin dynamics and cell migration processes by interacting with Huntingtin‐interacting protein 1 (Hip1) and Hip1‐related (Hip1R).^[^ ^14^ ^]^ In non‐small cell lung cancer (NSCLC), CLTB upregulation has been shown to enhance metastatic activity, trigger abnormal growth factor signaling, and correlate with poorer patient survival rates.^[^ ^15^ ^]^ However, the specific molecular mechanisms and biological functions of CLTB in HCC are yet to be fully elucidated.

This study comprehensively elucidates the dual regulatory role in the malignant progression of HCC through functional validation and sequencing‐based mechanistic analyses. It reveals that CLTB expression is elevated in HCC tissues compared to normal liver tissues, and this increase correlates with a lower survival rate in patients with HCC. CLTB and CLTB‐enriched sEVs enhance HCC cell proliferation and progression both in vitro and in vivo. Moreover, sEV‐CLTB induces angiogenesis, compromises vascular endothelial barrier integrity, and increases pulmonary vascular permeability. Mechanistically, CLTB enhances the cellular uptake of sEVs by coordinating the NF‐κB–proliferating cell nuclear antigen (PCNA) clamp‐associated factor (PCLAF/KIAA0101) axis, thereby promoting HCC progression. Meanwhile, sEV‐CLTB promotes vascular remodeling by binding to SH3 domain‐containing kinase‐binding protein 1 (SH3KBP1) and then inhibiting its degradation. This study is the first to identify CLTB as a central regulatory hub in HCC pathogenesis, connecting intracellular signaling and intercellular communication through dual molecular mechanisms.

## 2 Results

### 2.1 CLTB Expression Is Upregulated in HCC

**Figure 1:**
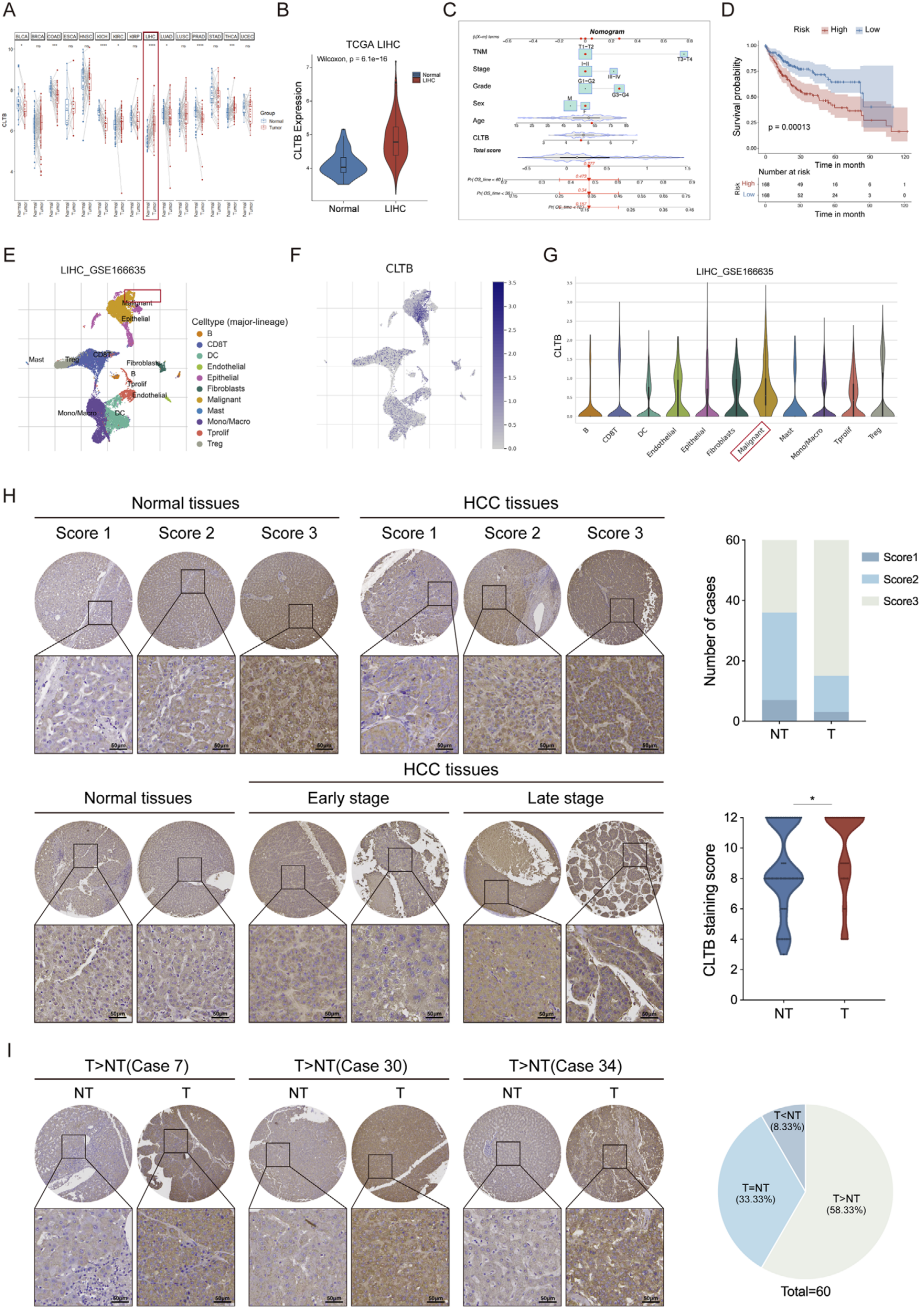
CLTB Expression Is Elevated in HCC. A) Expression of CLTB in TCGA cancerous tumors and surrounding tissues. B) TCGA datasets showed that CLTB expression was higher in LIHC tissues. C) Construction of a nomogram containing risk scores and other clinical characteristics for predicting survival in patients with LIHC. D) Kaplan–Meier overall survival curve analysis of patients with HCC for various risk groups. The low‐risk subgroup had a better prognosis (*p* < 0.001). E) Cell types identified by marker genes. F) CLTB expression levels in each cell subpopulation. G) Distribution of CLTB‐related activities in different cell types. H) Immunohistochemical results of CLTB in 60 paired HCC tissue microarrays. Representative images (left) and quantitative intensity score (right) analysis (n = 60). Scale bar: 50 µm. I) Representative images of CLTB overexpression in tumor tissue (left) and case composition ratio (right) (n = 60). Scale bar: 50 µm. The mean ± SEM is employed to present the data, *p < 0.05, **p < 0.01, ***p < 0.001 by t‐test (H).

In the pan‐cancer analysis of The Cancer Genome Atlas (TCGA) database, CLTB showed upregulated expression in various malignant tumors, with HCC being one of the most significantly upregulated tumors (**Figure** **1**A). Subsequent analysis combining TCGA and Gene Expression Omnibus (GEO) databases demonstrated significantly elevated CLTB expression in HCC tumor tissues compared to normal liver tissues (Figures 1B; S1A, Supporting Information). Receiver operating characteristic (ROC) analysis revealed that CLTB effectively discriminated HCC from normal tissues (AUC = 0.85, 95% CI: 0.798–0.901), suggesting potential diagnostic value (Figure S1B, Supporting Information). Kaplan–Meier analysis of the TCGA‐LIHC cohort stratified by median CLTB expression revealed a trend toward reduced overall survival (OS) in patients with high CLTB levels (Figure S1C, Supporting Information). A column‐line graphical model constructed based on CLTB expression levels and clinicopathological characteristics, including grade, stage, TNM, sex, and age, further validated that elevated CLTB expression substantially correlated with a worse prognosis (Figure 1C,D). Clustering and visualization of cell types through Uniform Manifold Approximation and Projection analysis, along with the cell marker database, identified 11 major cell subpopulations (Figure 1E) in which CLTB expression was predominantly localized to malignant cell clusters (Figure 1F,G).

CLTB expression was further analyzed in a clinical cohort of 60 patients with HCC. Immunohistochemical analysis showed significant differences in CLTB expression in normal and tumor tissues, with 75% (45/60) of tumor tissues showing intense positive staining for CLTB, which was markedly elevated compared to paracancerous tissues (Figure 1H). In addition, in 58.3% (35/60) of cases, the expression level of CLTB was significantly higher in tumor tissues than in matched paracancerous tissues (Figure 1I). Clinicopathological analysis revealed that elevated CLTB expression was significantly associated with cirrhosis (p = 0.047), but showed no correlations with sex, age, tumor characteristics (count, microsatellite status), histopathological features (Edmonson‐Steiner grade, TNM stage), viral hepatitis status (HBV/HCV), and serum AFP levels. The patients’ demographic and clinicopathological characteristics are shown in Table S1 (Supporting Information).

### 2.2 CLTB Enhances the Proliferation, Migration, Invasion, and sEV Uptake of HCC Cells

**Figure 2:**
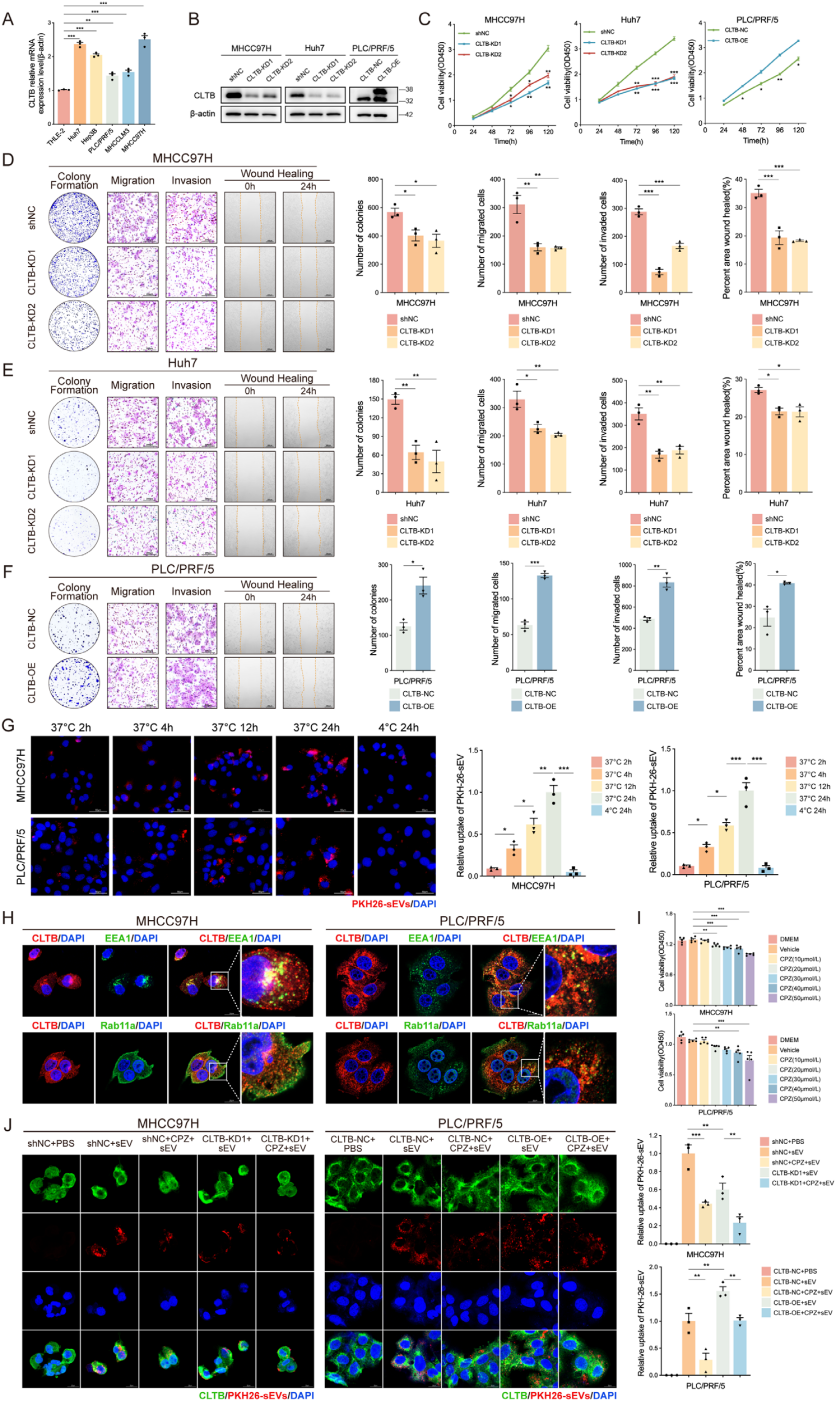
CLTB Enhances the Proliferation, Migration, Invasion, and sEV Uptake of HCC Cells. A) CLTB expression in HCC cells and THLE‐2 cells (n = 3). B) Western blot analysis of MHCC97H, Huh7, and PLC/PRF/5 to determine the efficiency of CLTB knockdown and overexpression (n = 3). C) Cell‐counting kit 8 (CCK‐8) was used to measure the viability of MHCC97H, Huh7, and PLC/PRF/5 stably transfected cells (n = 3). Colony formation, migration, invasion, and scratch wound healing assays were performed in MHCC97H D), Huh7 E), and PLC/PRF/5 F) cells after stable transfection (n = 3). Scale bar: 200 µm. G) Confocal microscopy was used to view PKH26‐labeled sEVs (red) co‐incubated with MHCC97H and PLC/PRF/5 cells at several temperatures and times (n = 3). Scale bar: 50 µm. H) Immunofluorescence detection of co‐localization of CLTB with EEA1 and Rab11a in PLC/PRF/5 and MHCC97H cells (n = 3). Scale bar: 20 µm. I) CCK‐8 assay was performed to determine the effective and non‐cytotoxic concentration of CPZ in PLC/PRF/5 and MHCC97H cells (n = 3). J) PKH26‐labeled sEV uptake was compared between CLTB‐KD1/shNC (MHCC97H) and CLTB‐OE/NC cells (PLC/PRF/5) with or without CPZ treatment (n = 3). The fluorescence intensity of PKH26 in each cell was measured using ImageJ software to quantify the absorption efficiency. The mean ± SEM is employed to present the data, *p < 0.05, **p < 0.01, ***p < 0.001 by *t*‐test (F), one‐way ANOVA (A, D, E, H, I, J), and two‐way ANOVA (C).

Consistent with the significantly heightened expression of CLTB in HCC tissues, CLTB levels were low in the normal human liver immortalized cell line, THLE‐2, but substantially increased in HCC cell lines, especially in the metastatic MHCC97H cells (**Figure** **2**A). To clarify the biological role of CLTB in HCC, MHCC97H and Huh7 cell lines with stable CLTB knockdown, as well as a PLC/PRF/5 cell line overexpressing CLTB, were constructed (Figure 2B). Functional experiments demonstrated that CLTB knockdown reduced the proliferative viability, colony‐forming capacity, migration, and invasion of MHCC97H and Huh7 cells (Figures 2C–E; S2, Supporting Information). Conversely, CLTB overexpression significantly enhanced the proliferative vigor and malignant phenotype of PLC/PRF/5 cells (Figures 2C,F; S2, Supporting Information).

In HCC, the internalization process of sEVs, which are key mediators of intercellular communication, significantly influences tumor cell proliferation and migration. Prior studies have demonstrated that sEV uptake depends on the CME pathway,^[^ ^13^ ^]^ suggesting that CLTB may enhance its oncogenic properties by mediating sEV uptake. sEV uptake experiments showed that uptake of PKH26‐labeled sEVs by MHCC97H and PLC/PRF/5 cells increased in a time‐dependent manner after co‐incubation at 37 °C. Conversely, sEV uptake was markedly reduced in the group treated at 4 °C, indicating that the absorption process of sEVs was energy‐dependent (Figures 2G; S3, Supporting Information). Immunofluorescence colocalization analysis showed extensive CLTB colocalization with recycling endosomal marker Rab11a and early endosomal marker EEA1, indicating its involvement in controlling endocytosis (Figure 2H). sEV uptake was significantly reduced in CLTB‐knockdown MHCC97H cells, whereas CLTB‐overexpressing PLC/PRF/5 cells exhibited enhanced sEV uptake. After confirming that 10 µmol/L chlorpromazine (CPZ), a clathrin inhibitor, had no cytotoxic effect (Figure 2I), we assessed its impact on sEV uptake. Quantitative analysis revealed significant impairment of sEV uptake efficiency in CPZ‐treated cells, demonstrating that CLTB mediates sEV uptake through clathrin‐dependent endocytosis (Figure 2J).

### 2.3 CLTB Drives HCC Progression Through NF‐κB‐Mediated PCLAF Upregulation

**Figure 3:**
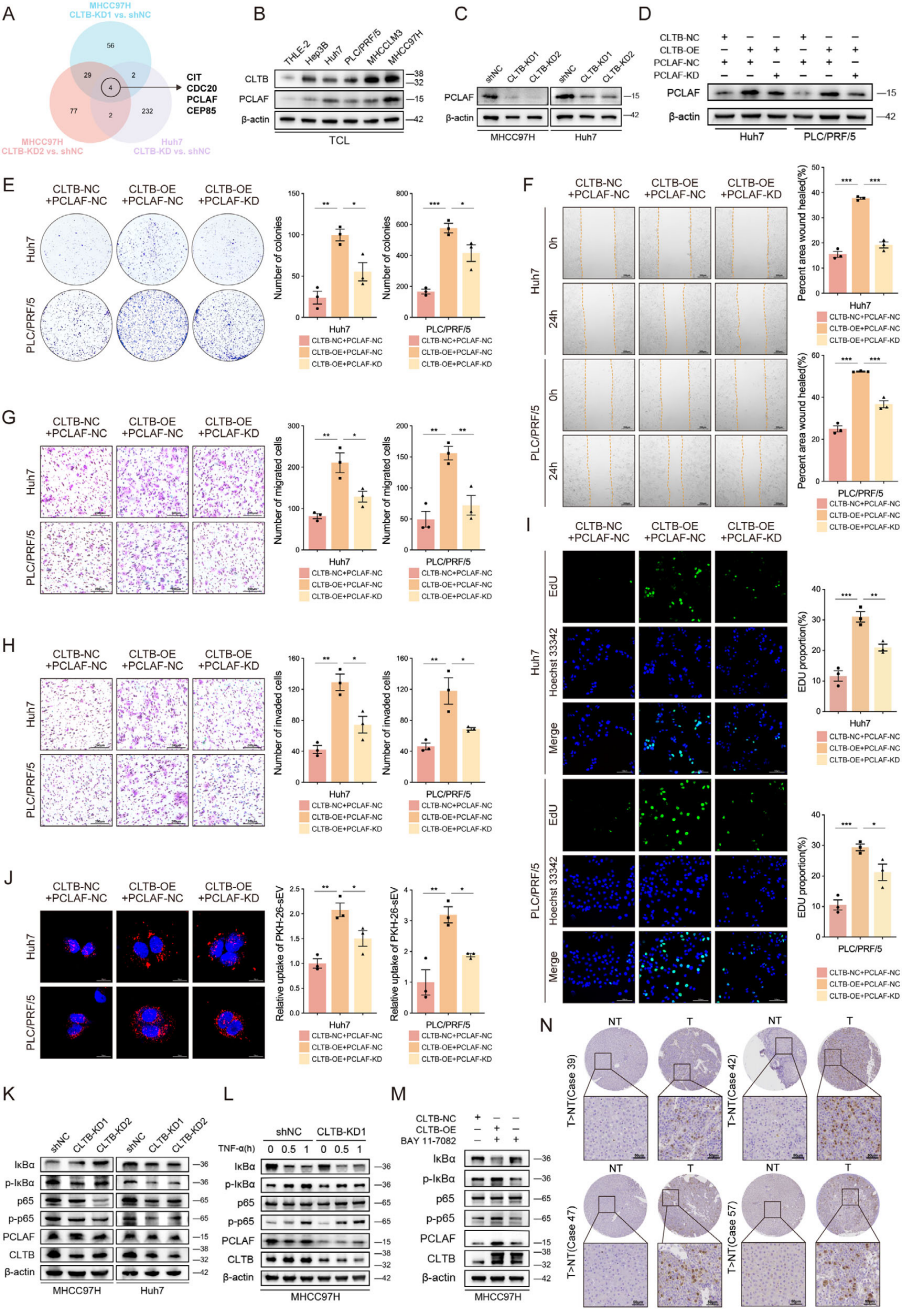
CLTB Promotes the Development of HCC via Activation of the NF‐κB–PCLAF Signaling Pathway. A) A Venn diagram showing four candidate proteins (CIT, CDC20, PCLAF, and CEP85) co‐downregulated in MHCC97H and Huh7 CLTB‐KD cells. Each comparison group included three biological replicates. B) PCLAF expression in HCC cell lines was examined using western blot (n = 3). C) PCLAF expression in Huh7 and MHCC97H shNC and CLTB‐KD cells as determined via western blot (n = 3). D) PCLAF expression in Huh7 and PLC/PRF/5 CLTB‐NC and CLTB‐OE cells transiently transfected with PCLAF‐NC and PCLAF‐KD plasmids as determined via western blot analysis (n = 3). After transfecting Huh7 and PLC/PRF/5 CLTB‐NC and CLTB‐OE cells with PCLAF‐NC and PCLAF‐KD vectors, tests for colony formation E), scratch healing F), migration G), invasion H), and cell proliferation I) were conducted (n = 3). Scale bar: 200 µm (F–H), 100 µm (I). J) Evaluation of the capacity of the indicated cells to uptake sEVs tagged with PKH26. Scale bar: 20 µm. K) NF‐κB signaling marker expression in MHCC97H/Huh7 shNC and CLTB‐KD cells as determined by western blot (n = 3). L) Western blot examination of TNF‐α‐stimulated NF‐κB signaling marker expression in MHCC97H shNC and CLTB‐KD cells (n = 3). M) Western blot analysis of NF‐κB signaling marker expression in MHCC97H CLTB‐NC, CLTB‐OE, and CLTB‐OE following blockage of BAY 11‐7082 (n = 3). N) PCLAF immunohistochemical staining of TMA, displaying representative images of paired HCC and normal tissues. Scale bar: 50 µm. The mean ± SEM is employed to present the data, *p < 0.05, **p < 0.01, ***p < 0.001 by one‐way ANOVA (E, F, G, H, I, J).

To clarify the molecular mechanisms underlying CLTB‐mediated regulation of HCC progression, we performed protein mass spectrometry analysis of CLTB‐knockdown (CLTB‐KD) and non‐targeting control (shNC) cells in MHCC97H and Huh7 lines. Intersection analysis identified four downregulated proteins: CIT, CDC20, PCLAF, and CEP85 (**Figure** **3**A). Leveraging clinical cohort data, we analyzed the expression correlations between CLTB and these key downstream proteins at both the transcriptomic (TCGA‐LIHC) and proteomic (CPTAC‐HCC) levels. Integrated multi‐omics analysis revealed that PCLAF exhibited a robust positive correlation with CLTB expression at both mRNA and protein levels, whereas the other candidate proteins (CIT, CDC20, and CEP85) showed no consistent or statistically significant correlations across datasets (Figure S4A–C, Supporting Information). Consistent with these clinical findings, quantitative reverse transcription polymerase chain reaction (qRT‐PCR) analysis of HCC cell lines further corroborated that PCLAF expression positively correlated with CLTB, whereas no significant associations were observed for the remaining proteins (Figure S4D, Supporting Information). Western blot further confirmed strong CLTB‐PCLAF co‐expression patterns, identifying PCLAF as a key downstream effector in CLTB‐mediated signaling pathways (Figure 3B). CLTB knockdown significantly inhibited PCLAF expression (Figure 3C), and CLTB overexpression upregulated PCLAF expression, which was suppressed upon PCLAF knockdown (Figure 3D). Functional rescue experiments demonstrated that PCLAF‐KD attenuated the pro‐oncogenic effects of CLTB, resulting in decreased cell proliferative vigor, colony‐forming ability, migration, and invasion capacity (Figures 3E–I; S5, Supporting Information), as well as sEV uptake efficiency (Figure 3J). In summary, CLTB and PCLAF are functionally interconnected and physiologically significant in HCC development.

Based on previous reports, the p50/RelB complex of the NF‐κB pathway acts as an upstream regulator, directly activating PCLAF transcription, promoting its expression, and driving nasopharyngeal carcinoma proliferation and metastasis.^[^ ^16^ ^]^ This prompted us to investigate whether CLTB mediates HCC progression by regulating aberrant PCLAF expression via the NF‐κB signaling pathway. Western blot examination of the expression of NF‐κB signaling markers after CLTB knockdown showed that CLTB gene silencing significantly inhibited the phosphorylation of IκBα and p65 (Figure 3K). Meanwhile, PCLAF expression was reduced in both the basal and induced states after TNF‐α stimulation in CLTB‐KD1 cells, suggesting that CLTB may regulate PCLAF stability or transcription (Figure 3L). Conversely, CLTB overexpression promoted IκBα degradation and sustained p65 phosphorylation. Thereby, the NF‐κB route, which was directly related to PCLAF overexpression, was activated, whereas the NF‐κB inhibitor, BAY 11‐7082, blocked this effect and reversed PCLAF upregulation, further establishing a causal relationship between CLTB‐mediated NF‐κB signaling and PCLAF expression (Figure 3M). To elucidate the mechanism by which the CLTB–NF‐κB axis regulates PCLAF expression, we investigated whether NF‐κB transcriptionally activates PCLAF. Bioinformatics analysis using the JASPAR database predicted high‐confidence binding sites for the NF‐κB subunit p50 within the PCLAF promoter region (Figure S6A, Supporting Information). Subsequent chromatin immunoprecipitation quantitative polymerase chain reaction (ChIP‐qPCR) in MHCC97H cells confirmed significant p50 binding activity at specific sites within the PCLAF promoter (Figure S6B, Supporting Information). Furthermore, luciferase reporter assays demonstrated binding of p50 to the predicted sites in the PCLAF promoter, validating its transcriptional regulatory capacity (Figure S6C, Supporting Information). Furthermore, clinical cohort analysis indicated significantly increased PCLAF immunoreactivity in tumor tissues relative to normal liver tissues, and the PCLAF expression pattern was congruent across patients exhibiting high PCLAF and CLTB expressions (Figure 3N). These findings depict a novel CLTB–NF‐κB–PCLAF regulatory axis and provide a mechanistic framework for understanding PCLAF dysregulation in CLTB‐related pathology.

### 2.4 HCC‐Derived CLTB‐Enriched sEVs Promote Tumor Progression In Vitro and In Vivo

**Figure 4:**
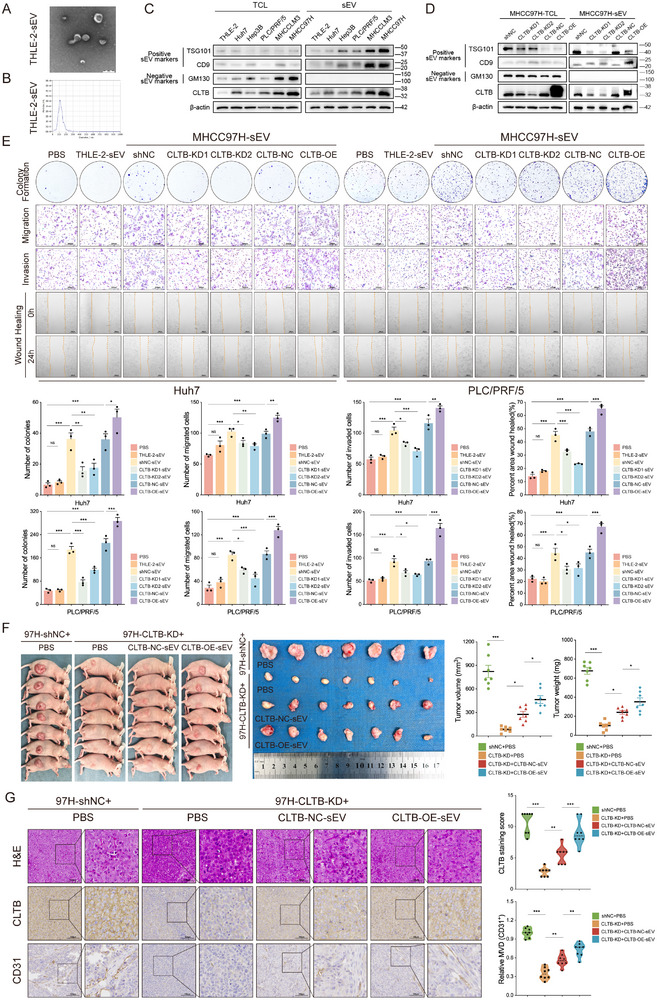
HCC‐Derived CLTB‐Enriched sEVs Promote Tumor Growth In Vitro and In Vivo. A) Analysis of the morphologic structures of the identified sEVs using transmission electron microscopy (TEM) (n = 3). Scale bar: 200 nm. B) After analyzing the particle size distribution of the indicated sEVs using nanoparticle tracking, the average particle size of the sEVs was reported (n = 3). C,D) Western blot was used to identify sEVs and analyze CLTB expression in total cell lysate (TCL) and sEVs. E) Colony formation, migration, invasion, and scratch wound healing assays were performed in Huh7 and PLC/PRF/5 cells treated with the indicated sEVs (n = 3). Scale bar: 200 µm. F) Representative images of mice (left) and tumors (middle) after subcutaneous injection of CLTB‐KD/shNC cells with PBS or sEVs. Tumor volume and weight were quantified (right) (n = 7). G) Tumor histopathology (HE stains) and protein expression of CLTB and CD31 (IHC) (n = 8). Scale bar: 100 µm. The mean ± SEM is employed to present the data, *p < 0.05, **p < 0.01, ***p < 0.001 by one‐way ANOVA (E, F, G).

CLTB was markedly expressed in HCC cells and HCC cell‐derived sEVs, with expression levels higher than those of the sEVs obtained from THLE‐2 cells. Isolated sEVs met stringent quality criteria through tripartite validation: sEV markers, transmission electron microscopy‐verified morphology, and nanoparticle tracking (**Figures** **4**A–C; S7, Supporting Information). To investigate CLTB‐enriched sEV functionality, we isolated sEVs from MHCC97H cells with CLTB‐stable knockdown (CLTB‐KD1 and CLTB‐KD2), shNC control, CLTB‐stable overexpression (CLTB‐OE), and vector control (CLTB‐NC), and verified their quality (Figures 4D; S7, Supporting Information). In vitro functional experiments indicated that CLTB‐NC‐sEV treatment enhanced clone formation, migration, and invasion of Huh7 and PLC/PRF/5 cells compared to the phosphate‐buffered saline (PBS) control, whereas CLTB‐OE‐sEVs further exacerbated these phenotypes. In contrast, CLTB‐KD1‐sEVs and CLTB‐KD2‐sEVs significantly suppressed these malignant phenotypes (Figure 4E). In the subcutaneous injection model, mice injected with CLTB‐KD cells and treated with CLTB‐NC‐sEVs showed increased subcutaneous tumor volume relative to the control group, with a more significant acceleration of tumor growth in the CLTB‐OE‐sEV‐treated group (Figures 4F; S8, Supporting Information). Immunohistochemical analysis demonstrated significantly reduced CLTB expression and CD31+ microvessel density (MVD) in CLTB‐KD group tumors compared to controls. Co‐injection of CLTB‐NC‐sEVs into CLTB‐KD‐derived tumors restored CLTB and MVD levels, with further enhancement observed upon CLTB‐OE‐sEV administration (Figure 4G).

### 2.5 sEV‐CLTB Interacts with SH3KBP1 to Facilitate Angiogenesis, Disrupt Vascular Endothelial Barrier Integrity, and Exacerbate Pulmonary Vascular Leakage

**Figure 5:**
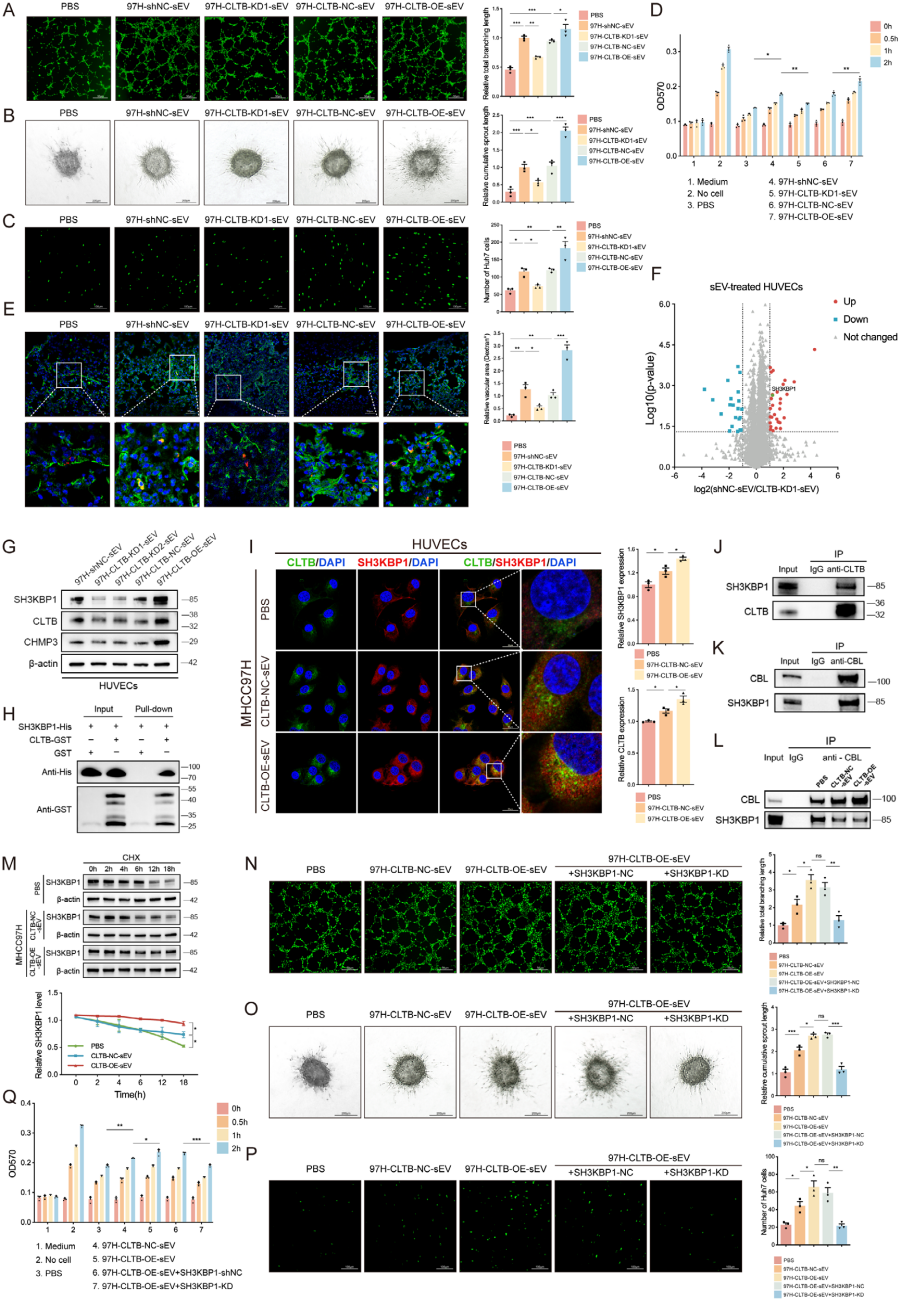
sEV‐CLTB Regulates Angiogenesis and Endothelial Barrier Dysfunction via SH3KBP1 Interactions. A) Tubule formation assay in HUVECs treated with indicated sEVs (n = 3). Scale bar: 50 µm. B) Spheroid sprouting assay in HUVECs treated with indicated sEVs (n = 3). Scale bar: 200 µm. C) Using the transendothelial invasion experiment, Huh7 cells treated with the proper sEVs were examined for penetration of the HUVEC monolayer (n = 3). Scale bar: 100 µm. D) TMR‐dextran leakage through HUVEC monolayers treated with indicated sEVs (n = 3). E) Texas‐Red dextran leakage areas in pulmonary vasculature (quantified assay) (n = 3). Scale bar: 50 µm. F) Proteomic profiling of HUVECs treated with shNC‐sEVs vs. CLTB‐KD1‐sEVs (volcano plot; fold change ≥2, *p*<0.05). Each comparison group included three biological replicates. G) Expression of SH3KBP1 and CHMP3 in HUVECs treated with the specified sEVs as determined via western blot analysis (n = 3). H) GST pulldown verified CLTB interactions with SH3KBP1. I) Immunofluorescence of CLTB (green) and SH3KBP1 (red) in HUVECs treated with CLTB‐NC‐sEVs and CLTB‐OE‐sEVs (n = 3). Scale bar: 20 µm. J) Co‐immunoprecipitation of CLTB in HUVECs (anti‐IgG vs. anti‐CLTB) (n = 3). K,L) Co‐immunoprecipitation with anti‐IgG or anti‐CBL antibodies in HUVECs (n = 3). M) SH3KBP1 protein stability in HUVECs treated with cycloheximide and sEVs (n = 3). N) Tubule formation assay in HUVECs transfected with SH3KBP1‐NC or SH3KBP1‐KD plasmids or treated with sEVs (n = 3). Scale bar: 50 µm. O) Spheroid sprouting assay in HUVECs under the same experimental conditions (n = 3). Scale bar: 200 µm. P) Transendothelial invasion assay quantifying Huh7 cell penetration through sEV‐ or SH3KBP1‐NC/KD‐treated HUVEC monolayers (n = 3). Scale bar: 100 µm. Q) TMR‐dextran permeability assay measuring vascular leakage in HUVECs. Fluorescence intensity in the lower chamber was quantified (n = 3). The mean ± SEM is employed to present the data, *p < 0.05, **p < 0.01, ***p < 0.001 by one‐way ANOVA (A, B, C, E, I, N, O, P), and two‐way ANOVA (D, M, Q).

Given the hypervascularity and metastatic propensity of HCC, we investigated the role of sEV‐CLTB in angiogenesis and vascular permeability. The findings indicated that CLTB‐NC‐sEVs markedly improved the tube‐forming capacity (**Figure** **5**A) and spherical germination (Figure 5B) of human umbilical vein endothelial cells (HUVECs), whereas CLTB‐OE‐sEVs further augmented these effects. In addition, we investigated whether sEV‐CLTB promoted cancer cell extravasation and induced vascular permeability. The permeability of HUVEC monolayers to green fluorescent‐labeled Huh7 cells (Figure 5C) and TMR‐dextran (Figure 5D) increased after exposure to CLTB‐NC‐sEVs and CLTB‐OE‐sEVs. In vivo, mice injected with CLTB‐OE‐sEVs exhibited a more extensive region of Texas Red dextran diffusion, indicative of pulmonary vascular permeability, compared to those administered control sEVs and CLTB‐KD1‐sEVs (Figure 5E).

To investigate the specific mechanisms of sEV‐CLTB‐induced angiogenesis and disruption of the vascular endothelial barrier, protein mass spectrometry analysis was conducted to examine MHCC97H shNC‐sEV‐ and CLTB‐KD1‐sEV‐treated HUVECs. Compared to CLTB‐KD1‐sEVs, 31 proteins demonstrated a minimum two‐fold upregulation (*p* < 0.05) in shNC‐sEV‐treated cells, with SH3KBP1 among the highest‐ranked differentially expressed proteins (Figure 5F). The GeneCards comprehensive database showed that, among the 31 upregulated proteins, only CHMP3 and SH3KBP1 were associated with sEV regulation. Further analysis revealed a positive correlation between SH3KBP1 and CLTB expression, whereas CHMP3 showed no correlation (Figure 5G). This indicated that SH3KBP1, which regulates clathrin‐dependent endocytosis, was a potential target modulated by CLTB. CLTB was shown to interact with SH3KBP1 in HUVECs via GST pulldown (Figure 5H) and immunoprecipitation (Figure 5J) experiments. Immunofluorescence results showed that CLTB and SH3KBP1 colocalized in HUVECs. Moreover, consistent with the results of flow cytometry, the expression rates of both CLTB and SH3KBP1 were markedly elevated upon treatment with CLTB‐OE‐sEVs, thus revealing a positive correlation between the expression of CLTB and SH3KBP1 (Figures 5I; S9, Supporting Information).

CBL has been reported to mediate the mono‐ubiquitination of SH3KBP1 through its interaction with SH3KBP1.^[^ ^17^ ^]^ We speculated that sEV‐CLTB interfered with the interaction between SH3KBP1 and CBL, thereby inhibiting the influence of CBL on the ubiquitination and degradation of SH3KBP1. Therefore, the interaction between SH3KBP1 and CBL in HUVECs was verified using immunoprecipitation (Figure 5K). Compared to the PBS treatment, the CLTB‐NC‐sEV treatment decreased the SH3KBP1‐CBL interaction, whereas CLTB‐OE‐sEVs further decreased this interaction (Figure 5L). HCC cell‐derived sEV‐CLTB bound to SH3KBP1, thereby alleviating the SH3KBP1‐CBL interaction and preventing CBL‐induced SH3KBP1 mono‐ubiquitination and degradation. To elucidate the regulatory impact of sEV‐CLTB on SH3KBP1 stability of proteins, cycloheximide was used to inhibit de novo protein synthesis, and degradation rates of SH3KBP1 were quantitatively assessed using time gradient studies. The results showed that SH3KBP1 was stabilized in the CLTB‐NC‐sEV‐treated group, whereas its degradation was further inhibited in CLTB‐OE‐sEV‐treated cells (Figure 5M).

To verify whether sEV‐CLTB regulation of HUVECs was mediated by SH3KBP1, we assessed whether SH3KBP1 knockdown impeded the effects of CLTB‐OE‐sEVs in HUVECs. sEV‐CLTB enhancement of HUVEC tube‐forming and germination ability was reversed after SH3KBP1 knockdown (Figure 5N,O). Additionally, both the transendothelial activity of Huh7 cells (Figure 5P) and TMR‐dextran leakage were reduced (Figure 5Q), confirming that SH3KBP1 was a key mediator in the regulation of endothelial permeability by sEV‐CLTB.

### 2.6 SH3KBP1‐Targeted Therapy and Blockade of CLTB Using a Clathrin Inhibitor Suppresses the Development of HCC Patient‐Derived Xenografts

**Figure 6:**
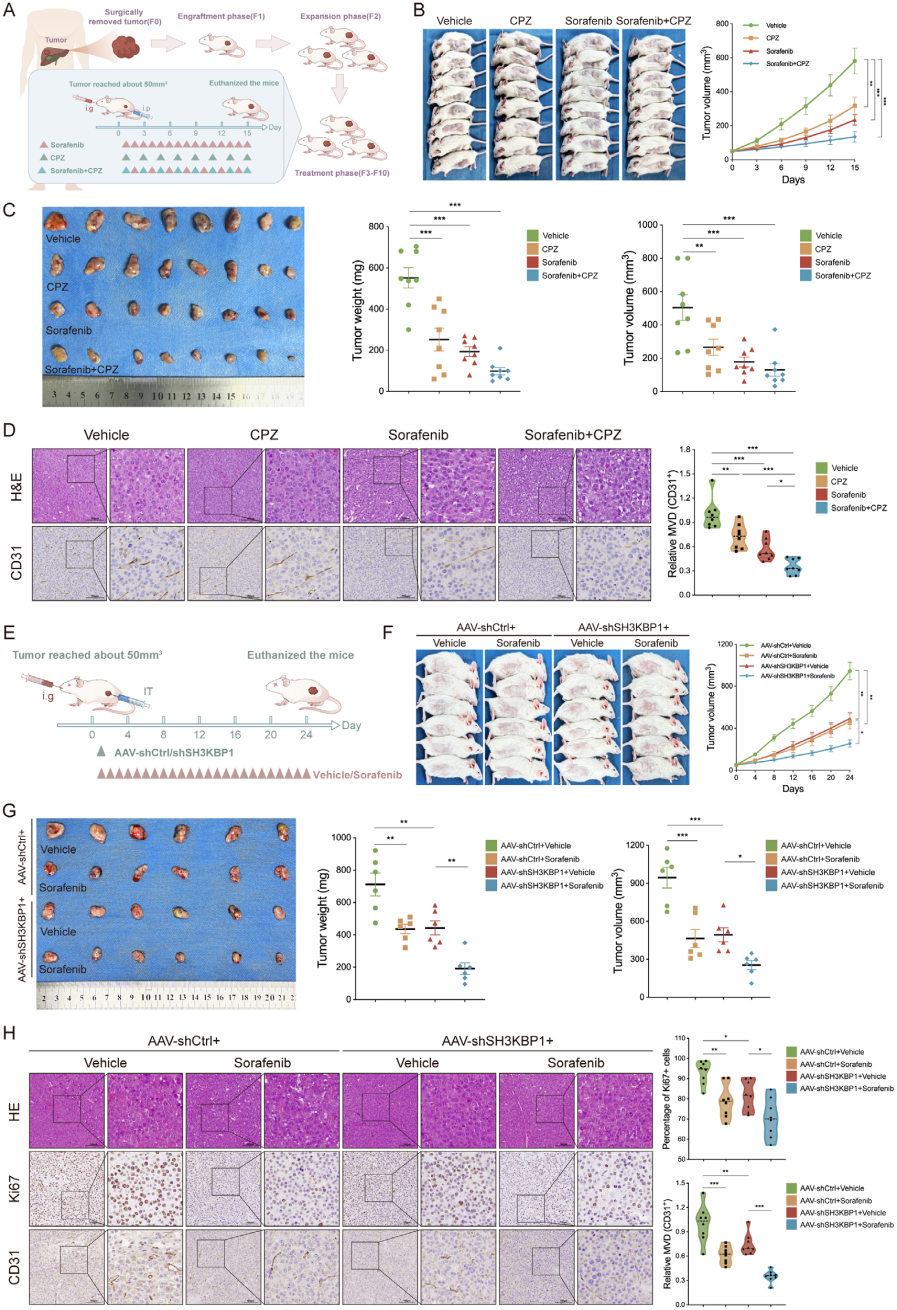
SH3KBP1‐Targeted Therapy and Blockade of CLTB Using a Clathrin Inhibitor Suppress the Development of HCC Patient‐Derived Xenografts. A) Schematic diagram of HCC PDX model construction and treatment group design. B) Representative images of mice (left) and tumor growth curves (right) across treatment groups at the experimental endpoint (n = 8). C) Tumor image at the end of the experiment (left). Tumor weight (middle) and volume (right) were measured (n = 8). D) HE staining was used to examine the histopathological appearance of the tumor, and IHC was used to find CD31 expression (n = 8). E) AAV‐shCtrl‐ and AAV‐shSH3KBP1‐treated PDX mice are shown schematically. F) Representative images of mice (left) and tumor growth curves (right) across treatment groups at the experimental endpoint (n = 6). G) Tumor (left) images at endpoint. Tumor weight (middle) and volume were quantified (right) (n = 6). H) Tumor histopathology (HE stains) and protein expression of CD31 and Ki67 (IHC) (n = 8). Scale bar: 100 µm. Scale bar: 100 µm. The mean ± SEM is employed to present the data, *p < 0.05, **p < 0.01, ***p < 0.001 by one‐way ANOVA (C, D, G, H), and two‐way ANOVA (B, F).

To identify the role of CLTB in driving HCC progression, we examined the therapeutic effect of CLTB drug inhibition using an HCC patient‐derived xenograft (PDX) model. Mice were treated with CPZ either alone or in combination with sorafenib (**Figure** **6**A). Both CPZ and sorafenib markedly impeded tumor growth compared to the untreated controls. The combined therapy demonstrated superior inhibitory effects compared to treatment with a single drug (Figure 6B,C). Immunohistochemical analysis revealed reduced expression levels of CD31+ MVD in the tumor tissues of either CPZ‐treated, sorafenib‐treated, or combined‐treated groups, indicating inhibition of tumor angiogenic pathways (Figure 6D).

To further validate SH3KBP1 as a therapeutic target, we assessed whether SH3KBP1 blockade inhibited tumor progression in a subcutaneous PDX mouse model. Sorafenib, a first‐line therapeutic agent for advanced inoperable HCC, was administered either alone or in conjunction with an adeno‐associated virus (AAV) targeting SH3KBP1 in PDX mice (Figure 6E). Both AAV‐shSH3KBP1 and sorafenib inhibited tumor progression, leading to smaller tumors than those in the vehicle group. The sorafenib and AAV‐shSH3KBP1 combination exhibited a more pronounced inhibitory effect than either monotherapy (Figure 6F,G). Immunohistochemical analyses revealed that CD31 and Ki67 levels were significantly reduced in tumors from mice treated with AAV‐shSH3KBP1 or sorafenib monotherapy, while the combined AAV‐shSH3KBP1 and sorafenib regimen exhibited a more pronounced reduction, indicating a synergistic inhibitory effect on angiogenesis and tumor proliferation via dual‐targeted intervention (Figure 6H).

## 3 Discussion

**Figure 7:**
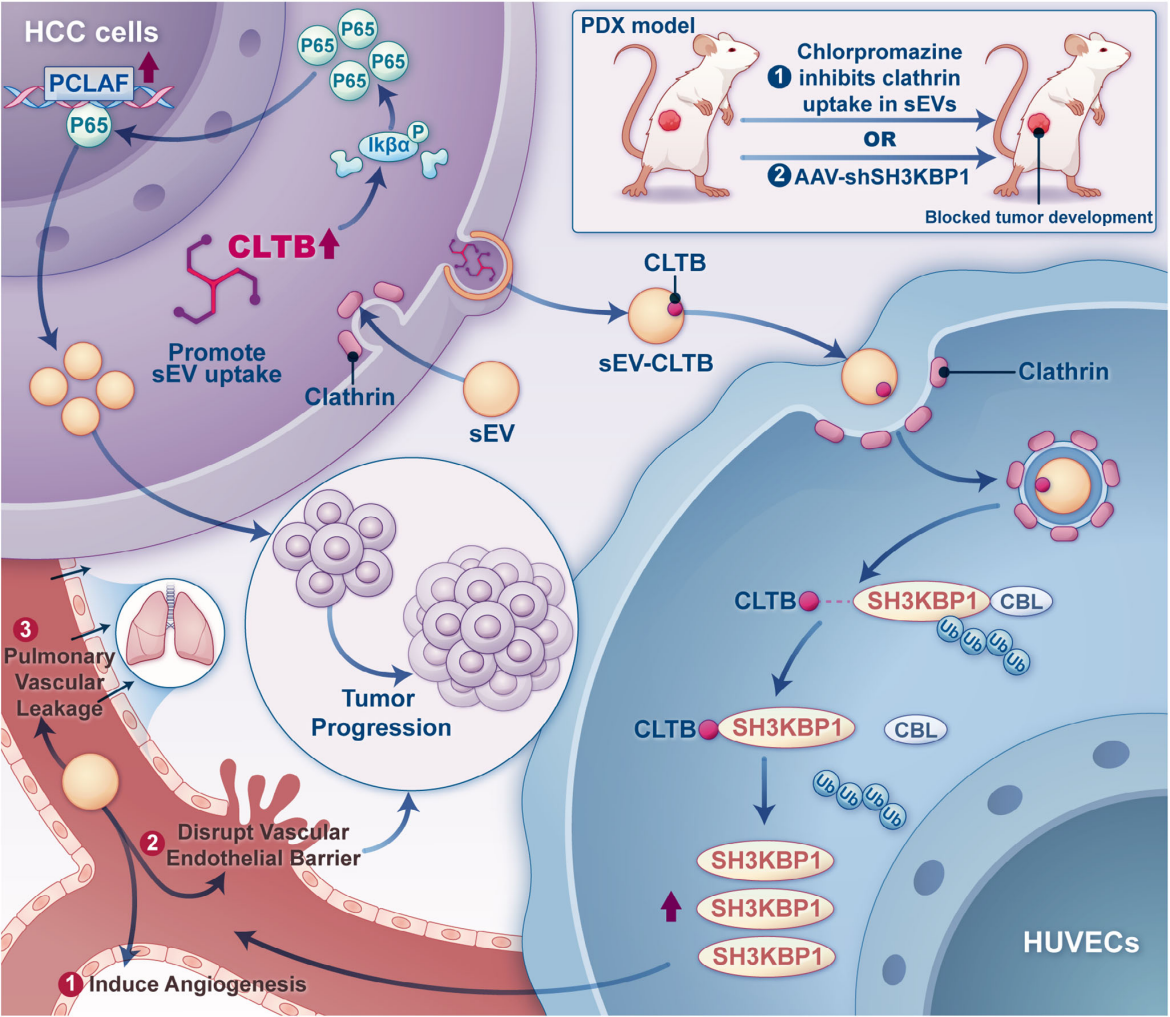
An Illustration Showing How CLTB Contributes to the Development of HCC. CLTB promotes HCC progression through the dual roles of intracellular signal transduction and intercellular sEV‐mediated communication. CLTB enhances sEV uptake via clathrin‐mediated endocytosis and promotes tumor progression by activating the intracellular NF‐κB‐PCLAF signaling axis. Concurrently, sEV‐CLTB secreted by HCC cells promotes endothelial angiogenesis, disrupts vascular integrity, and induces pulmonary vascular leakage by binding SH3KBP1 and then inhibiting SH3KBP1 degradation, thereby exacerbating tumor progression.

This study clarified the dual oncogenic function of CLTB in the development of HCC, revealing the multifaceted regulatory mechanisms it exerts through intracellular signaling and intercellular sEV communication (**Figure** **7**). We found that CLTB expression was strongly related to poor patient survival and was markedly raised in HCC tissues. CLTB promoted HCC cell invasion, migration, and proliferation, and enhanced the uptake of sEVs. Furthermore, sEV‐CLTB secreted by HCC cells exacerbated tumor progression by inducing angiogenesis, disrupting the integrity of the endothelial barrier, and increasing pulmonary vascular leakage. Mechanistically, CLTB regulated HCC development by activating the NF‐κB–PCLAF axis and stabilized its protein expression through an interaction with SH3KBP1, thereby remodeling the tumor microenvironment. These results provide a theoretical basis for therapeutic initiatives targeting CLTB and elucidate the pivotal function of CLTB in HCC.

CLTB, a critical subunit of the clathrin complex, plays a pivotal role in CME and cellular migration dynamics. CLTB has been implicated in cancer progression, with prior studies in NSCLC demonstrating that CLTB overexpression promotes metastatic potential via dysregulation of growth factor signaling pathways, a mechanism strongly associated with poor patient survival.^[^ ^15^ ^]^ Despite these insights in NSCLC, the role of CLTB in HCC remained unexplored. This study demonstrated significantly elevated CLTB expression in HCC tissues compared to adjacent non‐tumorous controls within a clinical cohort of 60 patients. CLTB significantly augmented the migration, invasion, and proliferation of HCC cells, with the highly metastatic cell line, MHCC97H, exhibiting notably elevated CLTB expression, indicating its pivotal role in HCC metastasis. Additionally, CLTB enhanced sEV uptake through CME, which in turn promoted HCC progression and invasive phenotypes. Inhibition of CLTB expression significantly decreased the internalization efficiency of sEVs, suggesting that CLTB is a key regulator of sEV uptake.

PCLAF, a PCNA‐interacting protein, promotes carcinogenesis through regulation of DNA damage repair and replication processes.^[^ ^18^, ^19^, ^20^ ^]^ While minimally expressed in normal tissues, PCLAF is markedly upregulated across multiple malignancies.^[^ ^21^, ^22^, ^23^, ^24^ ^]^ It has been identified as a significant oncogenic factor in various malignancies that promote pancreatic tumorigenesis,^[^ ^25^ ^]^ exhibiting a high level of expression in lung adenocarcinomas,^[^ ^26^ ^]^ and accelerating breast cancer development.^[^ ^27^ ^]^ Previous research has shown that the NF‐κB p50/RelB complex functions as an upstream regulator, directly activating PCLAF transcription, boosting its expression, and accelerating the growth and metastasis of nasopharyngeal carcinoma.^[^ ^16^ ^]^ To further explore how CLTB regulates sEV uptake, protein mass spectrometry analysis was performed on CLTB‐KD1 and shNC cells, and PCLAF was identified as a core differentially expressed protein. This finding prompted us to investigate whether CLTB mediated HCC progression by regulating aberrant PCLAF expression via the NF‐κB signaling pathway. Our results revealed that CLTB positively regulated the transcriptional activity of PCLAF through the modulation of the NF‐κB signaling pathway. As a central regulatory hub in inflammation‐related tumorigenesis, aberrant activation of NF‐κB mediates enhanced proliferation and inhibition of apoptosis in tumor cells.^[^ ^28^ ^]^ Experimental evidence suggests that CLTB maintains sustained activation of the NF‐κB signaling pathway by promoting IκBα and p65 phosphorylation modification, thereby upregulating the PCLAF expression level. This ultimately promotes the uptake of sEVs and accelerates HCC progression. Notably, PCLAF knockdown effectively reversed the CLTB‐induced malignant phenotype, confirming that the CLTB–NF‐κB–PCLAF axis plays a crucial regulatory function in the pathophysiology of HCC.

This study demonstrated CLTB enrichment in functional sEVs. Through sEV‐mediated intercellular communication, CLTB markedly increased the invasive metastasis of HCC cells, as demonstrated by in vitro cellular investigations and in vivo animal model studies. Given the important role of tumor‐derived sEVs in regulating angiogenesis and endothelial barrier, we explored the mechanism by which sEV‐CLTB increases tumor vascular invasion. HCC‐derived sEV‐CLTB increased pulmonary vascular leakage, compromised the integrity of the vascular endothelial barrier, and stimulated angiogenesis in HUVECs. The functional role of sEV‐CLTB in endothelial cell modulation suggests that tumor progression and metastasis are potentiated through sEV‐CLTB‐mediated angiogenesis, which facilitates cancer cell dissemination, induces pulmonary vascular permeability and tumor‐endothelial adhesion, and promotes tumor cell extravasation during metastatic colonization.

SH3KBP1 (also known as CIN85) is a multifunctional junction protein containing three SH3 structural domains that promotes tumor invasion and regulates actin remodeling.^[^ ^29^, ^30^, ^31^ ^]^ Previous studies have reported that CBL mediates the mono‐ubiquitination of SH3KBP1 through its interaction with SH3KBP1.^[^ ^17^ ^]^ CLTB interacted and co‐localized with SH3KBP1 in HUVECs, with a substantial positive connection in expression levels. This research further demonstrated that sEV‐CLTB inhibited the degradation of SH3KBP1 by binding to SH3KBP1, interfering with its interaction with CBL, thereby enhancing tubule formation and vascular leakage from endothelial cells. The significant association between elevated CLTB expression and poor prognosis underscores the potential of CLTB as a diagnostic biomarker. Sorafenib remains the first‐line systemic therapy for unresectable, advanced HCC. However, its tumor‐shrinkage efficacy is limited, with many patients developing resistance after prolonged treatment.^[^ ^32^, ^33^ ^]^ In this study, we investigated whether targeting CLTB or SH3KBP1 synergizes with sorafenib to inhibit HCC progression in PDX models. Combining either targeted approach with sorafenib effectively suppressed growth in CLTB‐expressing HCC PDXs and attenuated vascular abnormalities. This synergy suggests that targeting CLTB or its downstream effectors represents a promising strategy to overcome sorafenib resistance—a major challenge in advanced HCC management.

CLTB and clathrin light chain A (CLTA) are core subunits of the clathrin complex. Notably, previous studies have demonstrated that these two subunits share 60% amino acid sequence homology and exhibit differential expression levels across distinct cell types.^[^ ^11^ ^]^ Although both sEV‐CLTB and the previously reported sEV‐CLTA share functional attributes in promoting tumor angiogenesis and disrupting endothelial barrier integrity during HCC progression, the underlying mechanisms differ significantly.^[^ ^34^ ^]^ Importantly, these two studies collectively establish a comprehensive framework for understanding the role of the clathrin light chain family in HCC biology by elucidating—from complementary perspectives—their central functions in sEV‐mediated remodeling of the tumor microenvironment and metastasis.

Although this study provides significant insights into the dual oncogenic roles of CLTB in HCC progression, it has certain limitations. First, the clinical cohort analysis was conducted using 60 paired HCC tissues from a single medical center. Future multicenter studies incorporating multicenter patients with diverse etiological backgrounds (e.g., HBV/HCV infection status) are necessary to validate the universality of the diagnostic and prognostic value of CLTB. Second, although the PDX and subcutaneous tumor models provide valuable information, they do not fully demonstrate the complex interactions between HCC cells and the human tumor microenvironment, particularly immune cell interactions. Finally, the therapeutic potential of targeting CLTB or SH3KBP1 was demonstrated in preclinical models, but translational barriers such as drug delivery efficiency, off‐target effects, and long‐term toxicity in normal tissues were not addressed.

This study identifies CLTB as a key driver of HCC progression through dual mechanisms: the intracellular activation of the NF‐κB–PCLAF signaling axis facilitates sEV uptake and promotes tumor cell proliferation, while concurrently mediating vascular remodeling by stabilizing the SH3KBP1 protein through the inhibition of ubiquitination‐mediated degradation. CLTB overexpression correlates with poor prognosis, while preclinical models reveal that targeting CLTB or SH3KBP1 synergizes with sorafenib to suppress tumor growth and angiogenesis. These findings highlight CLTB as a therapeutic target and provide a rationale for dual‐pathway interventions in HCC.

## 4 Experimental Section

### Human Specimens

Sixty patients who underwent surgery for primary HCC at the Department of Hepatobiliary Surgery of the Second Affiliated Hospital of Harbin Medical University participated in this study. Paired tumor and adjacent non‐tumor tissues (collected >2 cm from the tumor margin) were obtained to construct tissue microarrays (TMAs). The Medical Ethics Committee of the Second Affiliated Hospital of Harbin Medical University approved the experimental protocol (ethical review approval No. YJSKY2024‐030). Written informed consent was obtained from all participants or their legally authorized representatives prior to the study. All human tissues were handled in compliance with the Declaration of Helsinki.

### Cell Culture

The HCC cell lines used in this experiment included Huh7 and MHCC97H (National Collection of Authenticated Cell Cultures, China); PLC/PRF/5 and Hep3B (Procell Life Science and Technology, China); and MHCCLM3 (Cellverse, China). In addition, the immortal human hepatocyte cell line THLE‐2 (Keycell, China) and HUVECs (Procell Life Science and Technology, China) were used. All cell lines were cultured according to the suppliers’ protocols. The experimental procedures followed accepted quality control guidelines, and every cell was regularly examined for mycoplasma contamination. Information on cell lines is presented in Table S2 (Supporting Information).

### Construction of Vectors and Establishment of Stable Clones

Lentiviral expression plasmids containing the target gene (overexpressed lentiviral vectors with C‐terminal Flag tags), and helper plasmids (HelpL1/L2/L3) were extracted using endotoxin‐free kits. Subsequently, 293FT cells were resuscitated and subcultured. Cells were inoculated in 10 cm gelatin‐coated dishes at a rate of 5 × 10⁶ cells/dish (liposome method), and the cell fusion reached 90–95% before transfection. On the day of transfection, the helper plasmid mixture and lentiviral expression plasmid were dissolved in Opti‐MEM preheated at 37 °C, then mixed with Opti‐MEM and Lipofectamine 2000 and incubated for 20 min to form a complex. The mixture was added dropwise to cells that had been replenished with fresh antibiotic‐free medium and incubated overnight at 37 °C. Virus supernatants were collected in several portions at 48 and 72 h after transfection. After concentration and titration, the virus was aliquoted in HBSS buffer. When cell confluence reached 70%, fresh complete medium was added. Lentivirus particles were then introduced at designated multiplicities of infection, followed by incubation at 37 °C for 6 h. The virus‐containing medium was subsequently replaced with fresh medium. After 72 h of transfection, clones with the highest expression levels were selected for puromycin drug screening. Cells that completed drug screening in 6‐well plates were further expanded. The sequences of oligonucleotides are provided in Table S3 (Supporting Information).

### Isolation and Validation of sEVs

EV‐depleted fetal bovine serum (FBS) was generated by ultracentrifugation of standard FBS (164210, Procell, China) at 100 000 × g for 18 h at 4 °C (Beckman Optima XPN‐100, SW32Ti rotor). After 72‐h cultivation in EV‐depleted FBS medium, the supernatant and sEVs were obtained by differential centrifugation. To remove cells and cellular debris, the conditioned media were first centrifuged at 3000 × g for 15 min, followed by centrifugation at 20 000 × g for 30 min. The conditioned media were filtered using a 0.22‐µm filter (SLGP033RB, Millipore, USA) to exclude large cellular debris and membrane structures. The post‐filtration supernatant was centrifuged at 4 °C, 100 000 × g for 70 min to produce a precipitate of crude extracted sEVs, which were subsequently resuspended in pre‐cooled PBS and re‐washed by centrifugation at 4 °C, 100 000 × g for 70 min. The precipitate was collected and resuspended in PBS for subsequent analyses. Western blot analysis was conducted to identify sEVs using positive and negative marker proteins. sEV size and concentration were measured using a Nanoparticle Tracking Analysis (NTA) system (ZetaView PMX 110, Particle Metrix, Germany) and its accompanying software (ZetaView version 8.04.02). Isolated sEV samples were appropriately diluted in 1X PBS buffer prior to measurement. The system was calibrated using 100 nm polystyrene nanoparticles. The characteristic cup‐shaped morphology of sEVs was detected using a transmission electron microscope (H‐7650, Hitachi, Japan). Specifically, samples were adjusted to an appropriate concentration, and ≈15 µL was applied onto copper grids for 1 min. Excess liquid was removed by blotting with filter paper. Subsequently, samples were stained with ≈15 µL of 2% (w/v) uranyl acetate for 1 min at room temperature (RT). After removing the stain by blotting with filter paper, grids were air‐dried and imaged.

### Pretreatment of Cells with sEVs

Cells were seeded in 6‐well plates at a density of 2 × 10^5^ per well and cultured overnight until confluence reached 70%. Following the removal of the supernatant, the plates underwent two PBS rinses. Cells were treated for 72 h with EV‐depleted complete medium containing 10 µg of isolated sEVs. Subsequently, functional tests and alternative assays were performed.

### Cell‐counting Kit 8 (CCK‐8)

Cells were seeded in 96‐well plates at 3 × 10^3^ cells per well in 100 µL complete medium and incubated for 24 h. Subsequently, the medium was discarded, and 100 µL of a 10% CCK‐8 (BS350B, Biosharp, China) solution was added to each well. The wells were then incubated for 4 h at 37 °C without light. The absorbance of each well was measured using a microplate reader (Infinite 200 PRO, TECAN, Switzerland) at 450 nm.

### Colony Formation Assay

Cells in the logarithmic growth phase were seeded into 6‐well plates at 1 × 10^3^ cells/well and cultured for 14 days. Colonies were fixed with 4% paraformaldehyde, stained with 0.1% crystal violet, and washed with PBS. Colonies containing more than 50 cells were counted using ImageJ (v1.54, NIH, USA).

### Cell Migration and Invasion Detection

Cells were resuspended in serum‐free medium and seeded into the upper chamber of Transwell inserts (8 µm pore size, Labselect) at 1 × 10^4^ cells/well. The lower chamber contained complete medium with 10% FBS. After incubation at 37 °C for 18 h, cells on the lower membrane were fixed with 4% paraformaldehyde, stained with crystal violet, imaged, and counted using ImageJ. In the invasion assay, transwell inserts were coated with Matrigel (1:8 dilution, 356234, Corning) and polymerized at 37 °C for 4 h. Cells were seeded as described above, and subsequent steps matched the migration protocol.

### Scratch Wound Assays

Cells in the logarithmic growth phase were seeded into 6‐well plates at 2 × 10^5^ cells/well. Cells were cultured for 24 h at 37 °C to achieve full monolayer cell confluence. A parallel line of scratches was created along the longitudinal axis of the plate using a 200 µL sterile pipette tip at a consistent pace. Images were obtained immediately following scratch development and at 24‐h intervals using the same observation points.

### EdU Cell Proliferation Assay

The EdU Cell Proliferation Test Kit (C0078S, Beyotime, China) was used to evaluate cell proliferation. At a density of 5 × 10^4^ cells per well, cells were seeded onto cell culture plates, and the appropriate intervention treatments were administered to the experimental groups. Before EdU labeling, a 2× EdU working solution (20 µM) was prepared by diluting 10 mM EdU stock 1:500 in pre‐warmed complete medium at 37 °C. Following the removal of the original medium, an equivalent amount of 2× working solution was introduced to achieve a final concentration of 10 µM, while ensuring the osmotic pressure of the culture system remained steady to mitigate interference with cellular activity. For two additional hours, the cells were incubated at 37 °C, and 4% EdU was used to stain the cells. Following the labeling, the cells were fixed for 15 min at RT using 4% paraformaldehyde. The EdU test was performed using the reaction mixture.

### qRT‐PCR

RNA was extracted using the conventional TRIzol reagent (15596‐026, Invitrogen, USA) following the manufacturer's protocol. Following extraction, the FastQuant RT kit (R223‐01, Vazyme, China) was used to reverse‐transcribe the RNA into cDNA. The CFX96 Touch Real‐Time PCR Detection System (Bio‐Rad, USA) with the AceQ Universal SYBR qPCR Master Mix (Q511‐02, Vazyme, China) was used to perform qRT‐PCR. The 2^−ΔΔCt comparative technique was used to determine the fold changes after normalizing the expression levels of all the targeted genes to those of *β*‐actin. Table S4 (Supporting Information) lists the primers used for qRT‐PCR.

### Western Blot

Protein samples were obtained by lysing cells with radioimmunoprecipitation assay (RIPA) buffer (P0013B, Beyotime, China) containing a protease inhibitor (P1005, Beyotime, China) and a phosphatase inhibitor (P1081, Beyotime, China). Proteins were quantified using a bicinchoninic acid (BCA) assay. Using a 12% sodium dodecyl sulfate polyacrylamide gel electrophoresis (SDS‐PAGE) pre‐cast gel (X15012Gel, ACE Biotechnology, China), 20 µg of total protein was electrophoretically separated and then wet transferred to a 0.22 µm PVDF membrane (03010040001, Roche, Switzerland). The membranes were blocked with 5% non‐fat milk in TBST for 1 h at RT, then incubated with primary antibodies at 4°C overnight. Following three 10‐min membrane washes with Tris‐buffered saline with Tween 20 (TBST), the membranes were incubated for 1 h at RT with goat anti‐rabbit/mouse secondary antibodies (ZB‐2301/ZB‐2305, ZSGB‐BIO, China) labeled with horseradish peroxidase (HRP). Chemiluminescent signals were detected using enhanced chemiluminescence (ECL) reagent (BL520A, Biosharp, China). Table S5 (Supporting Information) lists the primary antibodies used.

### sEV Uptake Assay

sEVs were fluorescently labeled with PKH26 Red Fluorescent Cell Linker (Sigma–Aldrich, USA). Following labeling, the sEVs were rinsed with PBS, purified using ultracentrifugation (100 000 × g, 4 °C, 70 min), and collected for future procedures. At a density of 5 × 10^3^ cells per well, cells were seeded into 96‐well plates and allowed to attach to the surface overnight. They were subsequently co‐incubated with 10 µg of tagged sEVs under varying time and temperature settings. After the sEVs were incubated, cells were washed with PBS. Flow cytometry assays were performed using the Flow NanoAnalyzer (Micro Plus, Apogee, UK) after digestion with trypsin. Immunofluorescence was fixed for 15 min at RT with 4% paraformaldehyde, and stained with 4',6‐diamidino‐2‐phenylindole (DAPI). Fluorescence images were acquired using a laser scanning confocal microscope, and cellular uptake efficiency was quantified by analyzing fluorescence intensity with ZEN Blue software (Carl Zeiss AG, Germany).

### Flow Cytometry

Single‐cell suspensions were adjusted to a concentration of 1 × 10⁵–1 × 10⁶ cells mL^−1^. Cells were pelleted by centrifugation at 300 × g for 5 min. After carefully aspirating the supernatant medium, the cell pellet was resuspended in 1 mL of 4% paraformaldehyde fixative and incubated at 4 °C for 20 min. Fixed cells were washed with PBS, pelleted by centrifugation, and subsequently permeabilized using a permeabilization reagent. Cells were then resuspended in 100 µL of assay buffer per tube, supplemented with 5 µL of concentrated goat serum to block non‐specific binding sites, and incubated at 37 °C for 10 min. For primary antibody staining, 1 µg of the respective isotype control antibody was added to the isotype control tube, and 1 µg of the specific primary antibody was added to the test sample tubes. All tubes were incubated at 37 °C for 45 min. Following incubation, cells were washed twice with 1 mL PBS. Secondary staining was performed by adding 5 µL of concentrated fluorophore‐conjugated anti‐rabbit antibody to all tubes except the blank control. Tubes were incubated at 37 °C for 40 min in the dark. Subsequently, cells were washed twice with 1 mL PBS and resuspended in 100 µL PBS. Samples were immediately analyzed using a Flow NanoAnalyzer (Micro Plus, Apogee, UK). Instrument settings, including gating and fluorescence compensation, were established using negative controls or single‐stained samples. Fluorescence histograms (FITC channel) were acquired for each sample, with a minimum of 10 000 events recorded per experimental group. Data analysis was performed using FlowJo software (FlowJo LLC).

### Animal Experiments

Animal experiments were approved by the Laboratory Animal Ethics Committee of the Second Affiliated Hospital of Harbin Medical University (Ethics No. YJSDW2024‐025) and conducted in compliance with the National Institutes of Health (NIH) Guide for the Care and Use of Laboratory Animals (8th Edition). The experiments were performed in compliance with the sanctioned protocol and applicable ethical and scientific standards.

### Establishment of PDX Models

To establish PDX tumor models, tumor specimens from surgically resected tumors were cut into 3 × 3 × 3 mm tissue blocks and transplanted subcutaneously into the right costal‐abdominal region of NYG mice (females, 4–6 weeks old) (Changsheng Biotechnology, China) via trocar needles. Tumors in F0 mice typically reached 800–1000 mm^3^ within 1–6 months. Xenografts were excised, fragmented into 3 × 3 × 3 mm pieces, and serially passaged into F1–F3 mice. Stable F3 PDX models were utilized for subsequent experiments.

### Combination Therapy with CPZ and Sorafenib in PDX Models

PDX models were established following the protocol described in the “Establishment of PDX Models” section. The mice with tumors (n = 32) were randomized into four treatment groups once the subcutaneous xenograft tumor volume reached 50 mm^3^ ± 10%: vehicle group (dimethyl sulfoxide [DMSO] in PBS, 100 µL/day, oral gavage; n = 8); CPZ (C0982, Sigma–Aldrich, USA) monotherapy group (10 mg/kg every 2 days, intraperitoneal injection; n = 8); sorafenib (Bay 43‐9006, MedChemExpress, USA) monotherapy group (30 mg/kg/day, oral gavage; n = 8); and combination group: CPZ (10 mg/kg, every 2 days, intraperitoneal injection) + sorafenib (30 mg/kg/day, oral gavage); n = 8. The tumor dimensions were measured regularly to monitor their development. The equation for calculating tumor volume was 0.52 × length × width^2^. After a 15‐day treatment duration, mice were euthanized by cervical dislocation under anesthesia, and the tumors were excised surgically. The weights and volumes of the tumors were documented, and the tumors were sectioned for further examination. Tumor dimensions were measured every 3 days using digital calipers, and body weight was recorded weekly to assess toxicity.

### Subcutaneously Injected Tumor Model

Female BALB/c nude mice (4–6 weeks old; Huachuang Sino, China) were selected, and a mixed suspension of 2 × 10^6^ 97H‐shNC or 97H‐CLTB‐KD cells, together with the specified sEVs, was administered subcutaneously. Tumor length and width were measured every 3 days using calipers. The trial was terminated on day 21 post injection. Upon completion of the trial, the tumors were excised after euthanasia of the mice, and their size and weight were quantified. Tumor volume was determined using the following formula: 0.52 × length × width^2^.

### Pulmonary Leakiness Model

Female BALB/c nude mice (4–6 weeks old) were chosen and pretreated with 20 µg of sEVs or an equivalent volume of PBS by tail vein injection 24 h before the experiment. Texas Red lysine‐fixable dextran (D1864, Invitrogen, USA) was delivered into the tail vein at a dosage of 100 mg/kg 2 h before the experiment. Concanavalin A (Con A) conjugate (C11252, Invitrogen, USA) was administered at a dose of 10 mg/kg via the tail vein 15 min before the experiment. At the start of the experiment, mice were sedated and perfused with PBS through the left ventricle as a substitute for circulating blood. Subsequently, perfusion was performed with 4% paraformaldehyde for 20 min. For 24 h, the removed lung tissues were dehydrated in a 30% sucrose solution. The frozen sections were 4 µm thick. DAPI (P0131, Beyotime, China) was used for nuclear counterstaining and examined using a Carl Zeiss LSM980 confocal microscope (Carl Zeiss AG, Oberkochen, Germany). Three portions of each lung were assessed using the ZEN Blue software, and three random regions within each section were imaged. The dextran area was measured using the ImageJ software.

### Recombinant AAV‐shRNA Virus Construction

AAV type 9 (AAV9) has great potential for current research and applications owing to its broad tissue penetration, low immunogenicity, and stable gene expression. In animal models, AAV9 can be used for the efficient transfer of target genes to investigate gene functions as well as disease pathogenesis. For in vivo studies, the SH3KBP1 shRNA sequence was packaged into the AAV9. The vector was transfected into AAV‐293 cells to obtain SH3KBP1 shRNA AAV (AAV‐shSH3KBP1). Viral vectors were screened for freedom from replicative viruses, lipopolysaccharides, and bacterial contaminants. Homologous AAV9 null virus (AAV‐shCtrl) was used as a blank control. The PDX model was constructed as described previously. The mice (n = 24) were randomized to four treatment groups once the subcutaneous explant tumor volume had grown to 50 mm^3^ ± 10%. Each group received an injection of either AAV‐Ctrl or AAV‐shSH3KBP1 (60 µL, 1.8 × 10^12^ vg mL^−1^, intratumoral injection; n = 6) on the first day of tumor development, concurrently administered with either vehicle (DMSO in PBS, 100 µL/day, oral gavage; n = 6) or sorafenib (30 mg/kg/day, oral gavage; n = 6). The tumor dimensions were measured regularly to monitor their development. The equation for calculating tumor volume was 0.52 × length × width^2^. Following a 24‐day course of medication, mice were euthanized by cervical dislocation under anesthesia, and the tumors were surgically removed. The tumors were cut for additional inspection, and the mass and size of the tumors were noted.

### Protein Mass Spectrometry

Proteins were extracted by boiling in SDT lysis buffer (4% SDS, 100 mM Tris‐HCl, pH 7.6) at 95 °C for 5 min and quantified using bicinchoninic acid assay. Using Coomassie Blue staining, proteins (20 µg) were observed after being resolved on 4%–20% SDS‐PAGE gels (180 V, 45 min). Following reduction (40 mM DTT, 37 °C, 1.5 h) and alkylation (20 mM IAA, in the dark, 30 min), filter‐aided sample preparation digestion was carried out. UA buffer and 25 mM ammonium bicarbonate (NH_4_HCO_3_) were used for washing. Overnight trypsin digestion (1:50 w/w) was carried out at 37 °C. C18 cartridges were used to desalt the peptides, which were vacuum‐concentrated and reconstituted in 0.1% formic acid. A Bruker timsTOF Pro mass spectrometer and Evosep One system were used for LC‐MS/MS analysis. A C18 column (15 cm × 150 µm, 1.9 µm) was used for separation, and the flow rate was 220 nL/min with 0.1% formic acid (A) and 0.1% formic acid in acetonitrile (B). PASEF acquisition (m/z 100–1700, 1/K0 0.75–1.35 Vs/cm^2^) was performed in positive ion mode (1.6 kV). MaxQuant 1.6.14 was used to identify and quantify the data. Each comparison group included three biological replicates.

### Immunofluorescence Analysis

Following inoculation on coverslips, the cells were fixed with 4% paraformaldehyde for 15 min and subsequently permeabilized with 0.1% Triton X‐100 (T8200, Solarbio, China) for 20 min at RT. Subsequently, they were treated in 5% bovine serum albumin (BSA) in PBS for 30 min at 37 °C. After the addition of primary antibodies, the mixture was maintained overnight at 4 °C in a humidified chamber. After being protected from light and incubated for 1 h at RT with fluorescent secondary antibodies labeled with Alexa Fluor™ 488 or 568, nuclei were counterstained with DAPI. Pictures were taken using the confocal microscope LSM980. Table S5 (Supporting Information) lists the primary antibodies used.

### Immunohistochemistry (IHC)

Human and murine tissues were fixed in 10% neutral buffered formalin for 24 h, dehydrated through graded ethanol, embedded in paraffin, and sectioned at 5 µm. The sections were stained with hematoxylin and eosin or by IHC. Xylene was used to deparaffinize the sections. Subsequently, samples were rehydrated using an alcohol gradient, followed by antigen retrieval and suppression of endogenous peroxidase activity. Subsequently, the paraffin slices or TMAs were blocked with 5% BSA for 1 h at RT. Primary antibodies were added to the prepared samples and incubated for the whole night at 4°C. For 1 h, the goat anti‐rabbit secondary antibody labeled with HRP was incubated at RT. High‐resolution digital photographs were captured via the Slide Scanning System SQS‐12P (XUYA Scientific, Beijing, China) and evaluated by two blinded pathologists. Staining intensity (0–3) and positive cell proportion (0–4) were multiplied to generate H‐scores (0–12). Pathologists analyzed CLTB expression and scored each tissue microarray core as follows: 0 for negative, 1 for weak positive, 2 for moderate positive, and 3 for strong positive, thereby quantifying the intensity. Table S5 (Supporting Information) lists the primary antibodies used.

### CHIP‐qPCR

Cells were crosslinked with 1% formaldehyde for 10 min by inverting flasks at RT and quenched with 0.125 M glycine for 5 min. The pellets were lysed in lysis buffer for 10 min. After centrifugation, the supernatant was discarded, and the pellet was lysed in lysis buffer and subjected to sonication. Sheared chromatin was incubated with primary antibody bound to the Protein A/G Agarose Beads (88802, Thermo Fisher, USA) overnight, followed by elution and reverse cross‐linking at 65 °C overnight. TE buffer was added to the DNA elution buffer, followed by RNase treatment (0.5 mg mL^−1^) at 37 °C for 30 min and proteinase K treatment (0.3 mg mL^−1^) at 51 °C for 1 h. Subsequently, the DNA was isolated and purified.

### Luciferase Assay

Seed HEK293T cells into a 24‐well plate and perform liposome transfection when the confluence reaches 70–80%. Mix 1.2 µg of plasmid DNA with 100 µL of serum‐free DMEM per well. Separately, mix 2 µL of transfection reagent with 100 µL of serum‐free DMEM. Incubate the mixture at RT for 20 min, then add the transfection complex after replacing it with fresh complete medium. Culture the cells at 37 °C and 5% CO_2_ for 48 h. For detection, cells were equilibrated to room temperature, and 500 µL of passive lysis buffer was added to each well for 10 min. The lysate was centrifuged, and 40 µL of the supernatant was transferred to a 96‐well plate. Immediately after adding 50 µL of firefly luciferase assay reagent (LAR II), luciferase activity was measured using a chemiluminescence reader. Subsequently, 50 µL of Stop & Glo reagent was added to measure sea squirt luciferase activity.

### Co‐Immunoprecipitation

After NP‐40 was used to lyse the cells, cell lysates were obtained, and the BCA assay was used to measure the protein level. Each reaction contained 500 µg lysate with 2 µg anti‐CLTB or anti‐CBL antibody, while negative controls used 2 µg isotype‐matched rabbit IgG (A7016, Beyotime). The incubation was conducted at 4 °C with gentle inversion overnight. Forty microliters of Protein A/G Magnetic Beads (HY‐K0202, MedChemExpress, USA) were individually aliquoted and washed five times with 1 mL NP‐40 lysis buffer (centrifugation at 2000 × g, 1 min). Proteins from the overnight incubation at 4 °C were extracted, introduced to the magnetic bead tubes, and subjected to rotating incubation at 4 °C for over 4 h. Protein‐magnetic bead complexes were adsorbed using a magnetic rack, the supernatant was gradually disposed of, and the beads were washed five times using NP‐40. Upon full aspiration of the supernatant, SDS protein loading buffer was added, followed by protein blot analysis to detect the target proteins. The positive control was 20 µg of whole cell lysate, whereas the negative control was IgG. Table S5 (Supporting Information) lists the primary antibodies used.

### GST Pull‐down

To conduct GST pull‐down assays, equal volumes (0.5 mg) of fusion proteins that were His‐tagged and GST‐tagged were combined and incubated on ice for 3 h. The mixture was then placed on beads containing Glutathione Sepharose 4B. Following five wash buffer washes, proteins were eluted using wash buffer mixed with 15 mM reduced glutathione. An anti‐His antibody (H1029, Sigma–Aldrich, USA) was used to detect the eluate after it was separated using 12% SDS‐PAGE and transferred onto a PVDF membrane.

### Tubule Formation Assay

Matrigel (100 µL/well; 356234, Corning) was thawed at 4 °C and pipetted onto pre‐chilled 24‐well plates using cold tips. Plates were polymerized at 37 °C for 30 min. To prevent the production of air bubbles, the plates were incubated in a thermostat set at 37 °C for 5 min. HUVECs with different pretreatments were digested and prepared in suspension, and each well was inoculated with 100 µL of 6.5 × 10^4^ cells. Fluorescence staining was performed using the live cell tracer, CMFDA (40721ES50, Yeason, China). Following a 6‐h incubation at 37 °C, the resultant tubules were examined and captured using a fluorescent microscope (Leica, Germany).

### Spheroid‐based Sprouting Assay

HUVECs (8 × 10^4^) were resuspended in 200 µL of M200 medium (M200500, Thermo Fisher, USA), and 1 mL of methylcellulose (m0512, Sigma–Aldrich, USA) was added to enhance sphere aggregation. A 25 µL drop of cell suspension was gently pipetted onto the culture dish using an 8‐channel pipette. The droplets were incubated upside down at 37 °C for 24 h. For collagen embedding, the spheroids were first rinsed with PBS, centrifuged for 5 min at 200 × g, and then resuspended in 2 mL of methylcellulose with 20% FBS. To prepare the collagen working solution, 0.5 mL of 10× medium 199 (M0650, Sigma–Aldrich, USA) was combined with 4 mL of the collagen (A1048301, Thermo Fisher, USA) stock solution on ice, and the pH was calibrated using 0.2 N NaOH until the indicator exhibited a pink hue. The collagen solution was combined with the spherical suspension in a 1:1 ratio to create a spherical collagen solution, which was stirred carefully to prevent the formation of air bubbles. After incubating 800 µL of the mixture per well for 30 min at 37 °C, 100 µL of M200 medium was added to each of the 24‐well plates. After 16 h of incubation at 37 °C, the spheres were fixed in 4% paraformaldehyde, and budding was observed and documented using an inverted microscope. The lengths of the sprouts created from the spheres were assessed using the ImageJ software.

### TMR‐Dextran Leakiness Assay

The upper chamber of a transwell plate with a hole size of 0.4 µm was seeded with HUVECs (2 × 10^5^) that had been pretreated with sEVs. A monolayer cell barrier formed after 4 h. The upper chamber was filled with 100 µL of TMR‐Dextran (HY‐158082C, MedChemExpress, USA) solution (20 mg mL^−1^), while the lower chamber was filled with 500 µL of Medium 200. Incubation occurred at 37 °C for 0, 30, 60, and 120 min, with the wavelength of 570 nm measured in the lower chamber at various time intervals, indicating the impact of the specified sEVs on the permeability of HUVEC monolayers.

### Trans‐Endothelial Invasion Assay

After being pretreated with sEVs, 2 × 10^5^ HUVECs were cultivated for 6 h to form a complete monolayer in the upper compartment of a transwell chamber with an 8 µm pore size. Huh7 cells were pre‐stained with a live cell tracer for 1 h, subsequently washed three times with PBS, and then a suspension of 2 × 10⁴ cells per 100 µL was formulated in a serum‐free medium. The cells were meticulously collected and introduced into the endothelium surface, followed by gentle agitation to achieve homogenization. 600 µL of complete medium with 10% FBS was introduced into the bottom chamber. After 16 h, paraformaldehyde fixation was performed, cells remaining in the upper chamber were carefully scraped away, and Huh7 cells that traversed the HUVECs barrier were examined and photographed using an inverted fluorescence microscope.

### Bioinformatics Analysis

RNA‐seq data in fragments per kilobase per million formats, along with the accompanying clinicopathological information for 414 patients, were obtained from the HCC project in TCGA database (https://portal.gdc.cancer.gov/) (TCGA‐LIHC). The HCC dataset, GSE14520, was obtained from the GEO database (https://www.ncbi.nlm.nih.gov/geo/). This study used the R package TCGAplot to analyze the correlation between CLTB expression levels across various cancers. A multivariate Cox regression model was constructed to evaluate the relationship of clinical markers (age, sex, grade, stage, and T stage) and CLTB expression with OS in patients with LIHC. The predictive significance of this line graph was evaluated via ROC curves.

### Statistical Analysis

The Wilcoxon rank‐sum test was used to assess the expression levels of CLTB in normal and malignant tissues, and statistical significance was determined (*p < 0.05, **p < 0.01, ***p < 0.001, ****p < 0.0001). Pearson's correlation analysis was used to evaluate the statistical correlations between CLTB and other parameters. The Kaplan–Meier technique and log‐rank test were used for survival analysis. Values were presented as means ± standard error of the mean (SEMs). The sample size (n) for each specific comparison was stated in the corresponding figure legend. Data were analyzed via one‐way analysis of variance (ANOVA) or Student's t‐test using GraphPad Prism V10.4.0 (GraphPad, San Diego, CA, USA). Statistical significance was set at *P* < 0.05.

## Conflict of Interest

The authors declare no conflict of interest.

## Supporting information



Supporting Information

## Data Availability

The data that support the findings of this study are available from the corresponding author upon reasonable request.
