# Supplementary material for: Clathrin Light Chain B Drives Hepatocellular Carcinoma Progression Through Dual Mechanisms: Small Extracellular Vesicle‐Mediated Angiogenesis and the NF‐κB–PCLAF Signaling Axis
Source: Adv Sci (Weinh). 2025 Aug 18;12(42):e08613. doi: 10.1002/advs.202508613 (PMC12622436; doi:10.1002/advs.202508613)
Supplement: Supplementary file 1 — Supporting Information [file ADVS-12-e08613-s001.docx]

Supporting Information

**Clathrin Light Chain B Drives Hepatocellular Carcinoma Progression Through Dual Mechanisms: Small Extracellular Vesicle-Mediated Angiogenesis and the NF-κB–PCLAF Signaling Axis**

*Xiaoke Sun^#^, Junchen Guo^#^, Ning Zhao, Guanghua Cui, Yun Bai, Meijuan Ding, Yi Xu***, Yu Yang**


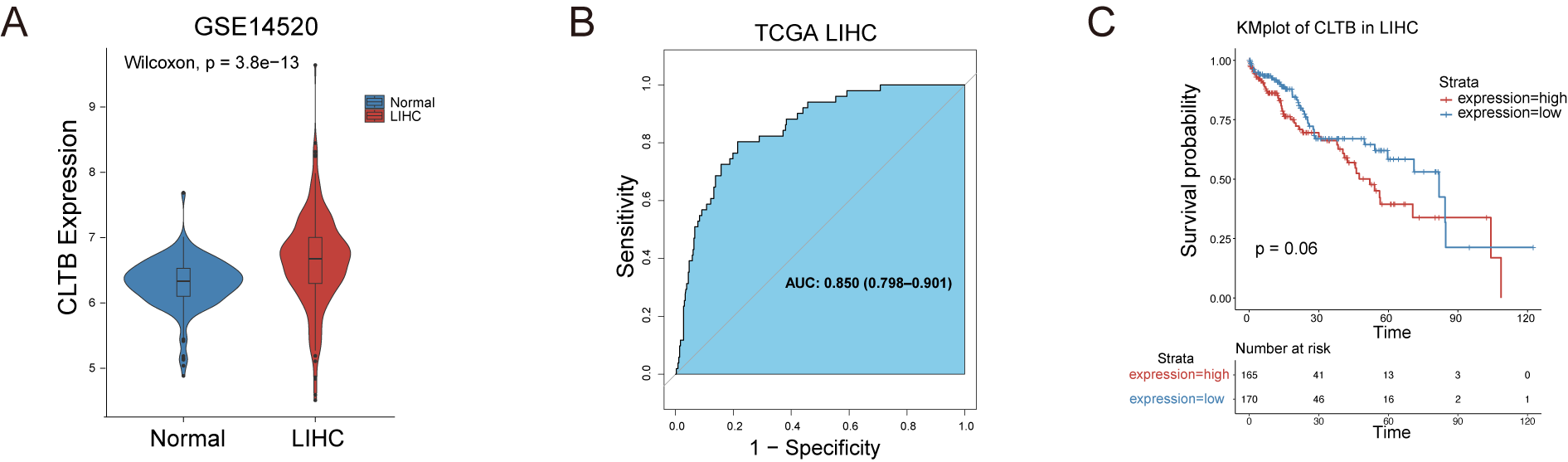


**Figure S1.** (A) The GSE14520 dataset showed that CLTB expression was higher in LIHC tissues. (B) ROC curve of CLTB expression in TCGA-LIHC. (C) Kaplan–Meier analysis demonstrated a trend toward lower OS in patients with HCC with high CLTB expression.


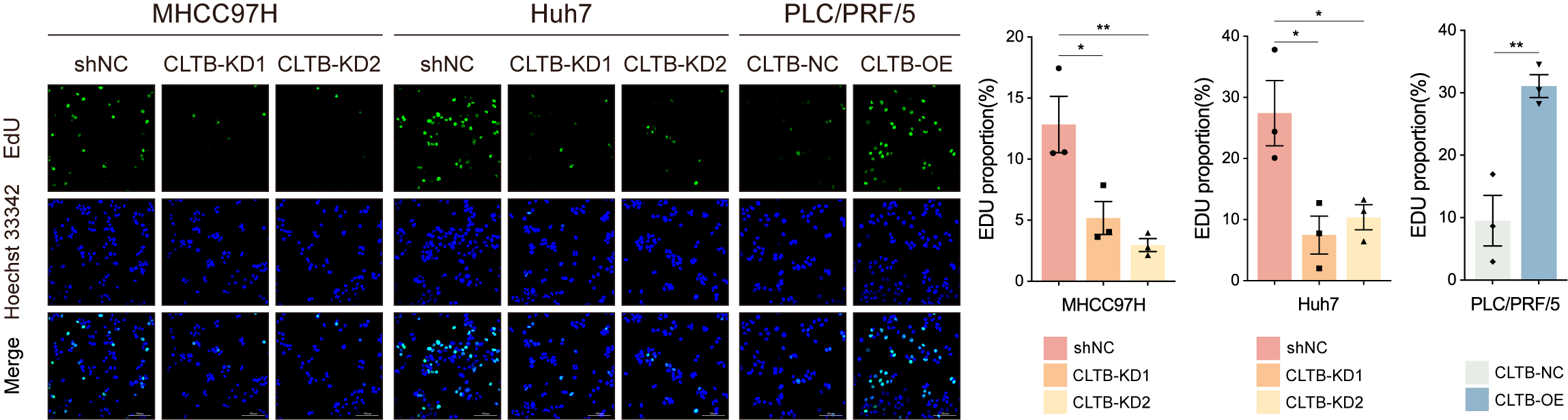


**Figure S2.** EdU cell proliferation assays were performed in MHCC97H, Huh7, and PLC/PRF/5 cells after stable transfection (n = 3). Scale bar: 100 μm. The mean ± SEM is employed to present the data, *p < 0.05, **p < 0.01 by one-way ANOVA.

**
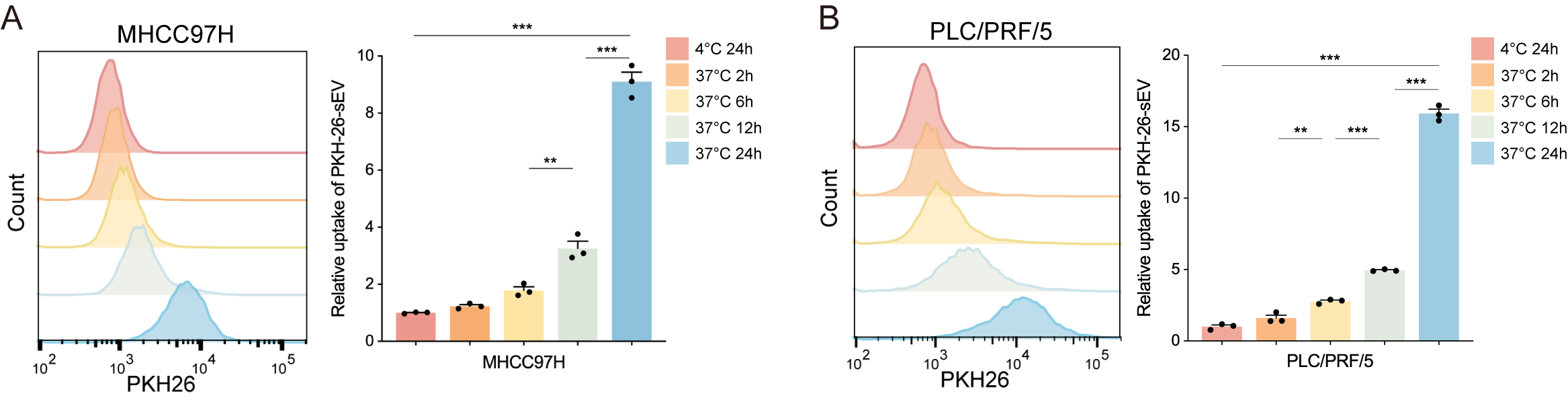
**

**Figure S3.** Flow cytometry was performed to detect sEV uptake after co-incubation of PKH26-labeled sEVs with MHCC97H and PLC/PRF/5 cells at different temperatures and times (n = 3). The mean ± SEM is employed to present the data, **p < 0.01, ***p < 0.001 by one-way ANOVA.

*
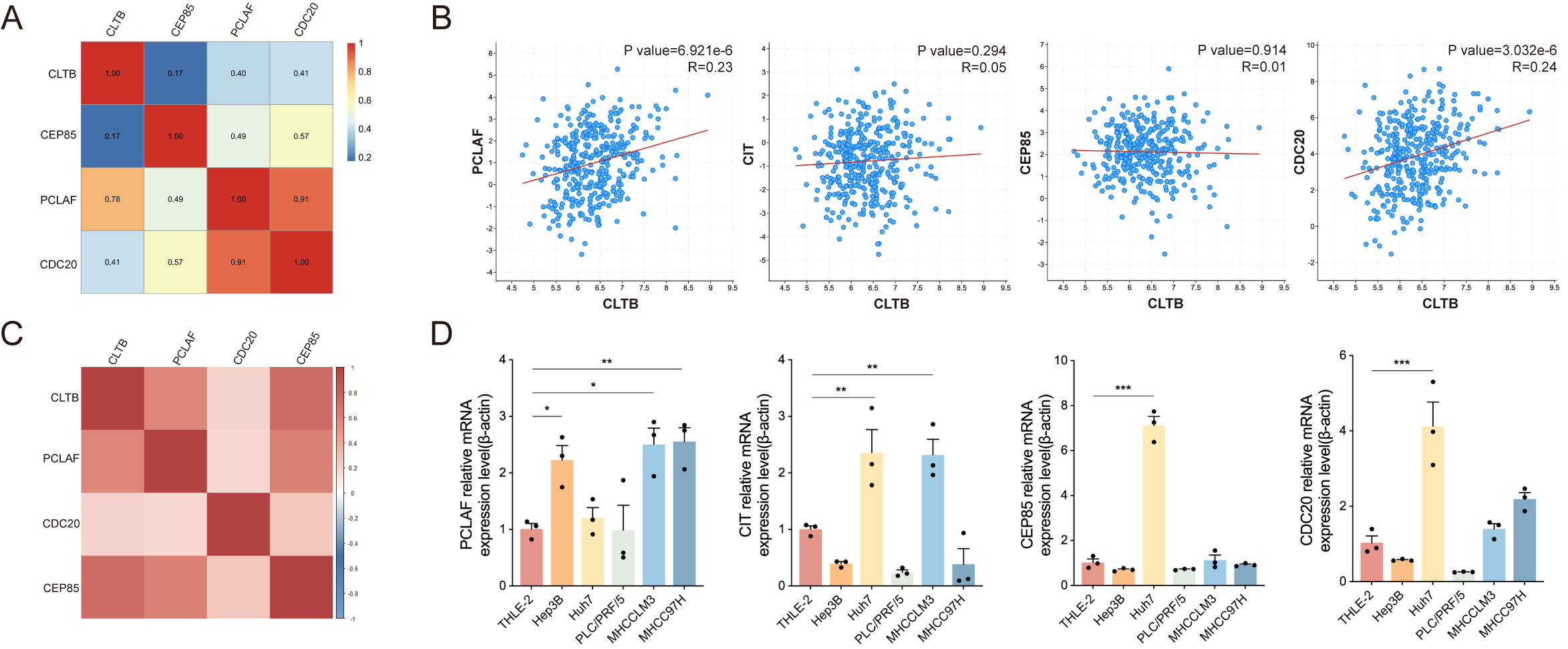
*

**Figure S4.** (A) Gene expression correlation matrix depicting expression correlation among four genes (CLTB, PCLAF, CDC20, and CEP85) in TCGA dataset. (B) Spearman correlation analysis of CLTB expression with PCLAF, CDC20, CIT, and CEP85. (C) Protein expression correlation matrix showing expression correlation among four proteins (CLTB, PCLAF, CDC20, and CEP85) in the CPTAC-HCC hepatocellular carcinoma proteogenomic dataset. (D) The levels of expression of CIT, CDC20, PCLAF, and CEP85 in HCC cell lines were compared using qRT-PCR (n = 3). The mean ± SEM is employed to present the data, *p < 0.05, **p < 0.01, ***p < 0.001 by one-way ANOVA.

**
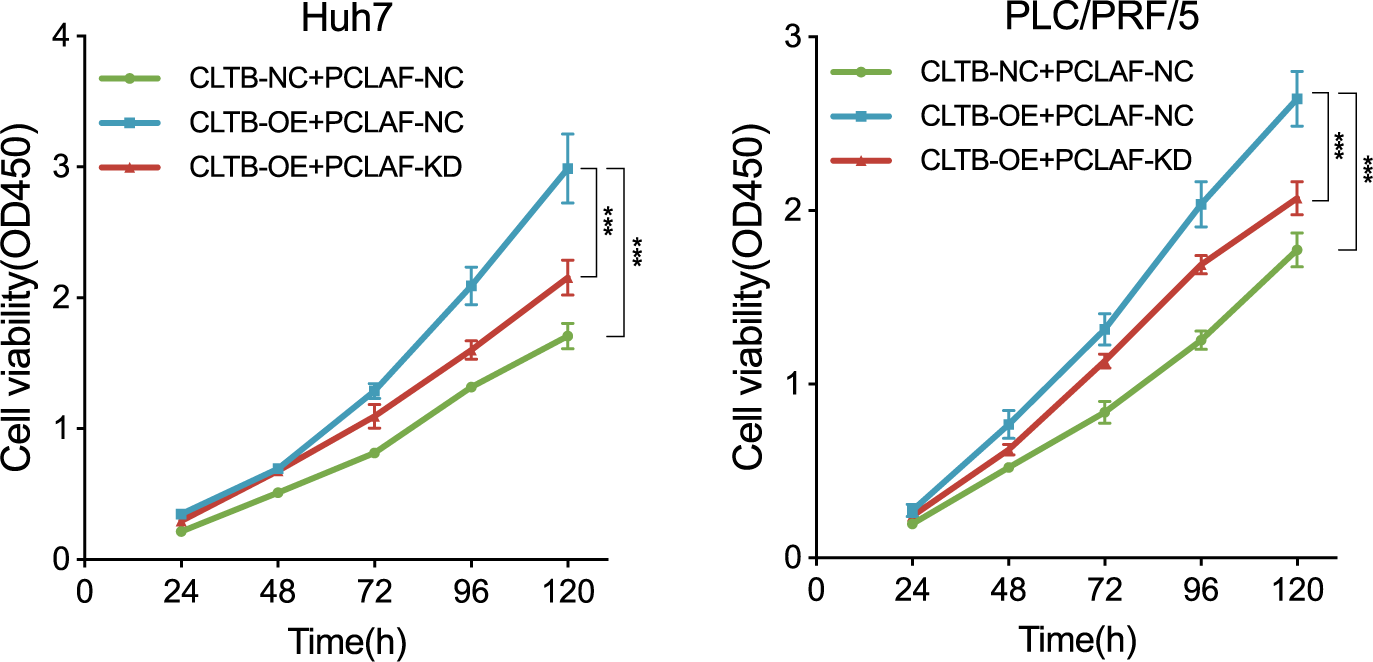
**

**Figure S5.** After transfecting Huh7 and PLC/PRF/5 CLTB-NC and CLTB-OE cells with PCLAF-NC and PCLAF-KD vectors, CCK-8 assays were performed (n = 3). The mean ± SEM is employed to present the data, ***p < 0.001 by two-way ANOVA.


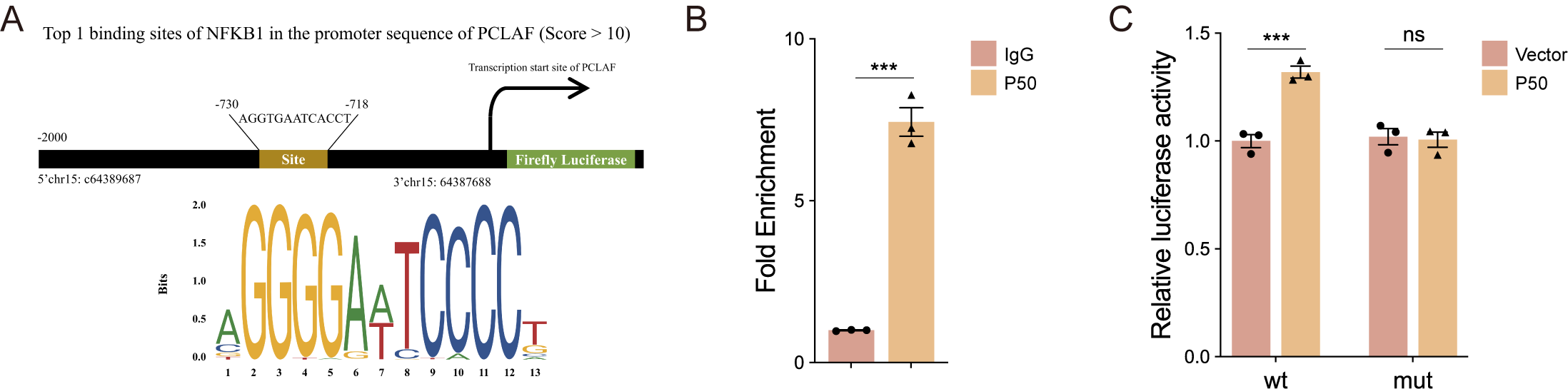


**Figure S6.** (A) Prediction of P50 binding sites within the PCLAF promoter region using the JASPAR database. (B) ChIP-qPCR analysis of P50 binding sites in the PCLAF promoter region in MHCC97H cells (n = 3). (C) Validation of P50 binding activity to the PCLAF promoter via luciferase reporter assay (n = 3). The mean ± SEM is employed to present the data, ***p < 0.001 by one-way ANOVA (B), and two-way ANOVA (C).

**
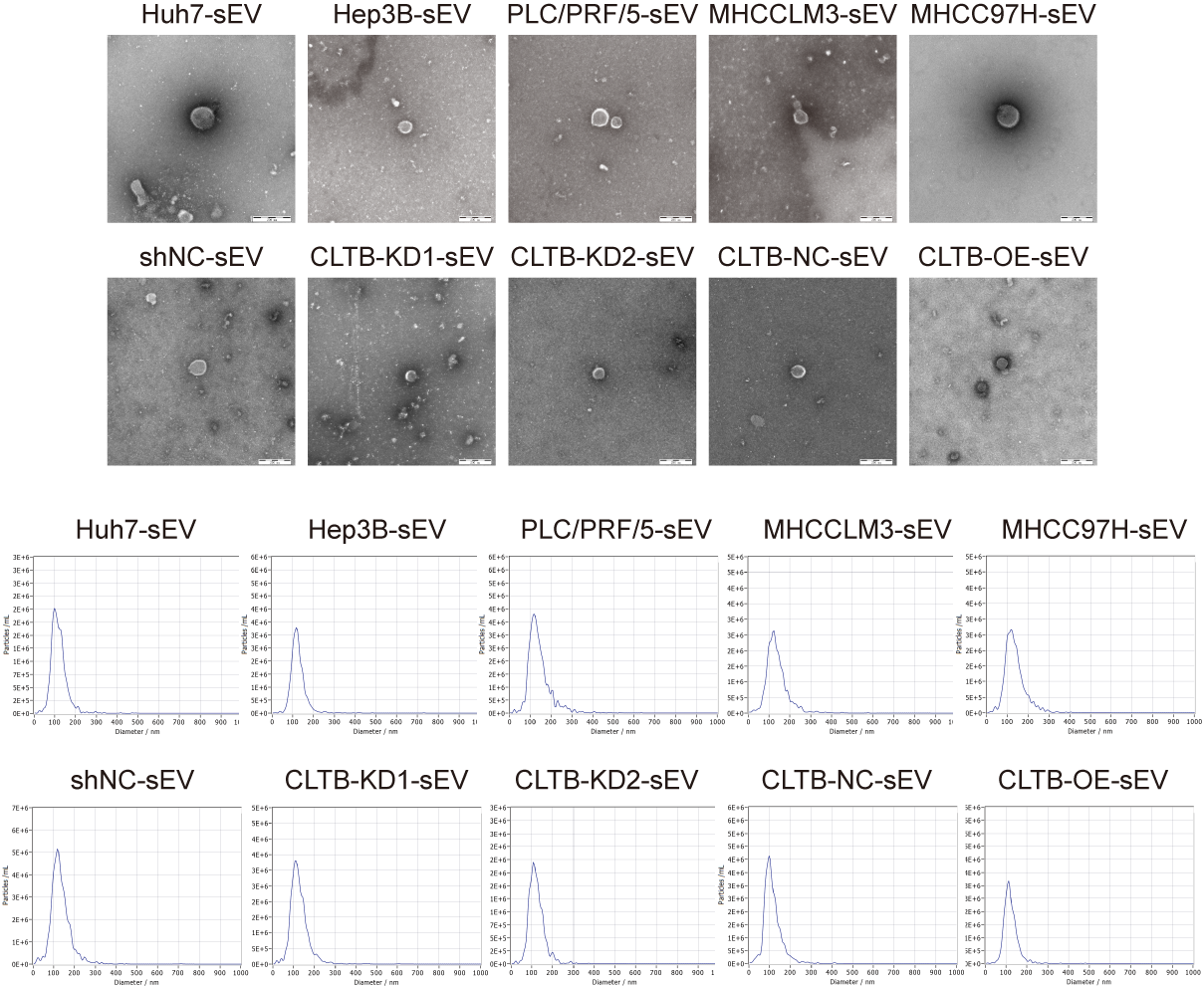
**

**Figure S7.** The morphology of the identified sEVs was analyzed using TEM (n = 3), and the average particle size was determined using nanoparticle tracking analysis (n = 3). Scale bar: 200 nm.


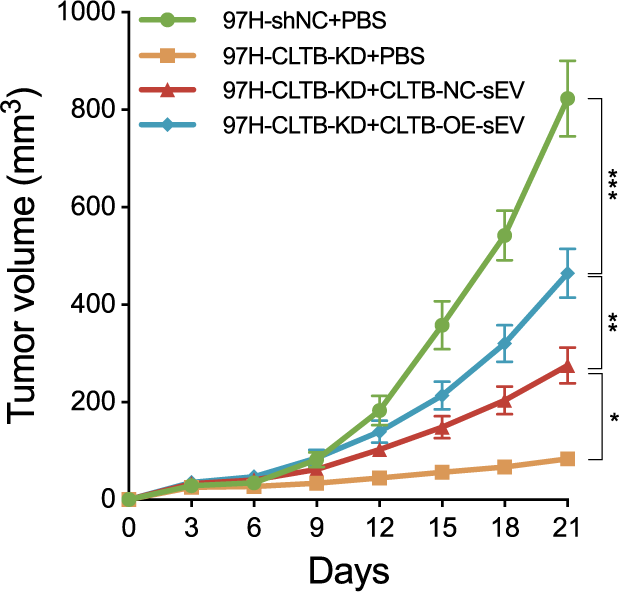


**Figure S8.** Growth curves of different subgroups in subcutaneous tumor formation experiments in nude mice (n = 7). The mean ± SEM is employed to present the data, *p < 0.05, **p < 0.01, ***p < 0.001 by two-way ANOVA.


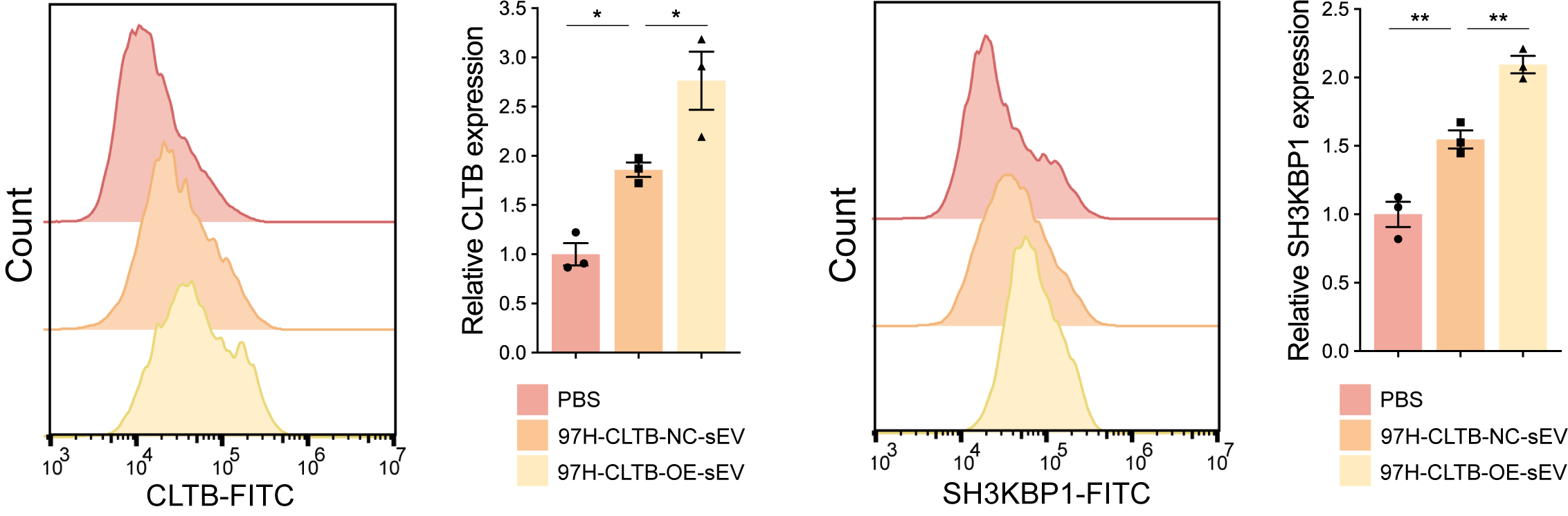


**Figure S9.** Expression of CLTB and SH3KBP1 in CLTB-NC-sEV- and CLTB-OE-sEV-treated HUVECs detected using flow cytometry (n = 3). The mean ± SEM is employed to present the data, *p < 0.05, **p < 0.01 by one-way ANOVA.

**Table S1.** **Associations between CLTB expression and clinicopathological characteristics of patients with HCC.**

| Clinicopathological | Total no. of | CLTB expression | | *p*-value | |  |
| --- | --- | --- | --- | --- | --- | --- |
| characteristics | patients | High (%) | Low (%) | |  | |
| Sex |  |  |  | | 0.671 | |
| Male | 54 | 28 (46.67%) | 26 (43.33%) | |  | |
| Female | 6 | 2 (3.33%) | 4 (6.67%) | |  | |
| Age (years) |  |  |  | | 0.552 | |
| <55 | 15 | 9 (15.00%) | 6 (10.00%) | |  | |
| ≥55 | 45 | 21 (35.00%) | 24 (40.00%) | |  | |
| Microsatellite |  |  |  | | 1.000 | |
| Positive | 9 | 5 (17.24%) | 4 (13.79%) | |  | |
| Negative | 20 | 12 (41.38%) | 8 (27.59%) | |  | |
| Liver cirrhosis |  |  |  | | **0.047** | |
| Present | 42 | 25 (41.67%) | 17 (28.33%) | |  | |
| Absent | 18 | 5 (8.33%) | 13 (21.67%) | |  | |
| Number of tumors |  |  |  | | 1.000 | |
| 1 | 53 | 27 (45.00%) | 26 (43.33%) | |  | |
| >1 | 7 | 3 (5.00%) | 4 (6.67%) | |  | |
| Tumor size |  |  |  | | 1.000 | |
| >5 cm | 31 | 15 (25.00%) | 16 (26.67%) | |  | |
| ≤5 cm | 29 | 15 (25.00%) | 14 (23.33%) | |  | |
| Lymph node metastasis |  |  |  | | 0.64 | |
| Positive | 6 | 2 (8.70%) | 4 (17.39%) | |  | |
| Negative | 17 | 9 (39.13%) | 8 (34.78%) | |  | |
| Vein invasion |  |  |  | | 0.237 | |
| Positive | 19 | 13 (27.08%) | 6 (12.50%) | |  | |
| Negative | 29 | 14 (29.17%) | 15 (31.25%) | |  | |
| TNM stage |  |  |  | | 0.748 | |
| I–II | 48 | 23 (38.33%) | 25(41.67%) | |  | |
| III–IV | 12 | 7 (11.67%) | 5 (8.33%) | |  | |
| Edmondson-Steiner grade |  |  |  | | 1.000 | |
| 1-2 | 8 | 5 (27.78%) | 3 (16.67%) | |  | |
| 3-4 | 10 | 6 (33.33%) | 4 (22.22%) | |  | |
| HBV infection |  |  |  | | 0.175 | |
| Positive | 36 | 21 (36.21%) | 15 (25.86%) | |  | |
| Negative | 22 | 8 (13.79%) | 14 (24.14%) | |  | |
| HCV infection |  |  |  | | 1.000 | |
| Positive | 9 | 4 (6.90%) | 5 (8.62%) | |  | |
| Negative | 49 | 25 (43.10%) | 24 (41.38%) | |  | |
| Serum AFP |  |  |  | | 0.765 | |
| >35 ng/mL | 15 | 7 (12.50%) | 8 (14.29%) | |  | |
| ≤35 ng/mL | 41 | 22 (39.29%) | 19 (33.93%) | |  | |

At p < 0.05, data in bold show statistical significance.

**Table S2. Information on cell lines.**

| **Name** | **Supplier** | **Cat no.** | **Authentication test method** |
| --- | --- | --- | --- |
| Human immortal hepatocyte cell line THLE-2 | Keycell, China | RRID: CVCL_3803 | Mycoplasma negative. Validated using both PCR and qPCR. Authenticated using STR. |
| Human HCC cell line Huh7 | National Collection of Authenticated Cell Cultures, China | RRID: CVCL_0336 | Mycoplasma negative. Validated using both PCR and qPCR. Authenticated using STR. |
| Human HCC cell line MHCC97H | National Collection of Authenticated Cell Cultures, China | RRID:  CVCL_4972 | Mycoplasma negative. Validated using both PCR and qPCR. Authenticated using STR. |
| Human HCC cell line MHCCLM3 | Cellverse, China | RRID: CVCL_6832 | Mycoplasma negative. Validated using both PCR and qPCR. Authenticated using STR. |
| Human HCC cell line PLC/PRF/5 | Procell Life Science and Technology, China | RRID: CVCL_0485 | Mycoplasma negative. Validated using both PCR and qPCR. Authenticated using STR. |
| Human HCC cell line Hep3B2.1-7 | Procell Life Science and Technology, China | RRID: CVCL_0326 | Mycoplasma negative. Validated using both PCR and qPCR. Authenticated using STR. |
| Human umbilical vein endothelial cells (HUVECs) | Procell Life Science and Technology, China | RRID: CVCL_2959 | Mycoplasma negative. Validated using both PCR and qPCR. Authenticated using STR. |

**Table S3. Sequences of oligonucleotides used in this study.**

| **shRNAs** | **Sense (5′–3′)** |
| --- | --- |
| shNC | CCTAAGGTTAAGTCGCCCTCG |
| shCLTB-KD#1 | GAGCTGGATGCTGCATCTAAG |
| shCLTB-KD#2 | AGCTGCTACCCACAGCCTATT |
| **CLTB-OE** | ATGGCTGATGACTTTGGCTTCTTCTCGTCGTCGGAGAGCGGTGCCCCGGAGGCGGCGGAGGAGGACCCGGCGGCCGCCTTCCTGGCCCAGCAGGAGAGCGAGATTGCAGGCATAGAGAACGACGAGGGCTTCGGGGCACCTGCCGGCAGCCATGCGGCCCCCGCGCAGCCGGGCCCCACGAGTGGGGCTGGTTCTGAGGACATGGGGACCACAGTCAATGGAGATGTGTTTCAGGAGGCCAACGGTCCTGCTGATGGCTACGCAGCCATTGCCCAGGCTGACAGGCTGACCCAGGAGCCTGAGAGCATCCGCAAGTGGCGAGAGGAGCAGAGGAAACGGCTGCAAGAGCTGGATGCTGCATCTAAGGTCACGGAACAGGAATGGCGGGAGAAGGCCAAGAAGGACCTGGAGGAGTGGAACCAGCGCCAGAGTGAACAAGTAGAGAAGAACAAGATCAACAACCGGATCGCTGACAAAGCATTCTACCAGCAGCCAGATGCTGATATCATCGGCTACGTGGCATCCGAGGAGGCTTTCGTGAAGGAATCCAAGGAGGAGACCCCAGGCACAGAGTGGGAGAAGGTGGCCCAGCTATGTGACTTCAACCCCAAGAGCAGCAAGCAGTGCAAAGATGTGTCCCGCCTGCGCTCGGTGCTCATGTCCCTGAAGCAGACGCCACTGTCCCGC |
| **siRNAs** | **Sense (5′–3′)** |
| siNC | UUCUUCGAACGUGUCACGUTT |
| siPCLAF | GUGCUUGGUUCUUCCACCUTT |
| siSH3KBP1 | CACUGAGAGAAAACAUGAATT |

**Table S4. Sequences of primers used for qRT-PCR.**

| Primer names | Primer sequences |
| --- | --- |
| CLTB | Forward: GCTGCTACCCACAGCCTATT |
|  | Reverse: TAGACTGAGAGGAGGCGTGA |
| PCLAF | Forward: GTGCTTGGTTCTTCCACCTCTG |
|  | Reverse: CCTTTTTGCCACTTGGGAGTTGG |
| CIT | Forward: AGCACAAGGCTGAGATTCTCGC |
|  | Reverse: CTCGTTCAGTCTCCAGCTTCTG |
| CDC20 | Forward: CGGAAGACCTGCCGTTACATTC |
|  | Reverse: CAGAGCTTGCACTCCACAGGTA |
| CEP85 | Forward: CTTAGAGCCAGCACAGTGGATC |
|  | Reverse: CTGTGGACTTGCAGTTCGCTCT |
| β-actin | Forward: CACCATTGGCAATGAGCGGTTC |
|  | Reverse: AGGTCTTTGCGGATGTCCACGT |
| PCLAF Primers used for CHIP-qPCR | Forward: TGGTGGTGCGTGCCTGTA |
|  | Reverse: CTGCAACCTCCGCCTTCC |

**Table S5.** **Information on the antibodies used in this study.**

| **Antibody** | **Supplier** | **Cat** | **Application** |
| --- | --- | --- | --- |
| CD9 | Abcam | ab236630 | WB |
| TSG101 | Proteintech | 28283-1-AP | WB |
| GM130 | Abcam | ab52649 | WB |
| CLTB | Proteintech | 10455-1-AP | WB, IF |
| β-Actin | Proteintech | 66009-1-Ig | WB |
| PAF15 | Cell Signaling Technology | 81533 | WB, IF |
| IκBα | Proteintech | 10268-1-AP | WB |
| phosphorylated IκBα | Proteintech | 82349-1-RR | WB |
| p65 | Proteintech | 10745-1-AP | WB |
| phosphorylated p65 | Proteintech | 82335-1-RR | WB |
| CIN85 | Proteintech | 12132-1-AP | WB, IF |
| VPS24 | Proteintech | 5472-1-AP | WB |
| EEA1 | Proteintech | 68065-1-Ig | IF |
| Rab11a | Proteintech | 67902-1-Ig | IF |
| CLTB | Boster | A09071-1 | IHC, FCM |
| CD31 | Proteintech | 28083-1-AP | IHC |
| Ki67 | Proteintech | 27309-1-AP | IHC |
| CBL | Proteintech | 25818-1-AP | IP |
| SH3KBP1 | Boster | A02835-1 | FCM |
